# A generic approach towards afterglow luminescent nanoparticles for ultrasensitive in vivo imaging

**DOI:** 10.1038/s41467-019-10119-x

**Published:** 2019-05-02

**Authors:** Yuyan Jiang, Jiaguo Huang, Xu Zhen, Ziling Zeng, Jingchao Li, Chen Xie, Qingqing Miao, Jie Chen, Peng Chen, Kanyi Pu

**Affiliations:** School of Chemical and Biomedical Engineering, Nanyang Technological University, 70 Nanyang Drive, Singapore, 637457 Singapore

**Keywords:** Nanoparticles, Fluorescent probes, Nanoscale materials, Polymers

## Abstract

Afterglow imaging with long-lasting luminescence after cessation of light excitation provides opportunities for ultrasensitive molecular imaging; however, the lack of biologically compatible afterglow agents has impeded exploitation in clinical settings. This study presents a generic approach to transforming ordinary optical agents (including fluorescent polymers, dyes, and inorganic semiconductors) into afterglow luminescent nanoparticles (ALNPs). This approach integrates a cascade photoreaction into a single-particle entity, enabling ALNPs to chemically store photoenergy and spontaneously decay it in an energy-relay process. Not only can the afterglow profiles of ALNPs be finetuned to afford emission from visible to near-infrared (NIR) region, but also their intensities can be predicted by a mathematical model. The representative NIR ALNPs permit rapid detection of tumors in living mice with a signal-to-background ratio that is more than three orders of magnitude higher than that of NIR fluorescence. The biodegradability of the ALNPs further heightens their potential for ultrasensitive in vivo imaging.

## Abstract (web summary)

Afterglow luminescence is used to reduce background noise and increase sensitivity; however, biocompatible afterglow materials are limited. Here, the authors report on an approach to turn standard optical agents into afterglow nanoparticles and demonstrate the application in tumour imagining in vivo.

## Introduction

Optical imaging that utilizes photon–electron interactions to decipher biological processes has grown into an indispensable tool in biomedical research and clinical practice^1^. Complementary to tomographic modalities such as magnetic resonance imaging (MRI), computed tomography (CT), and positron emission tomography (PET), optical imaging has the unique advantages of high spatial-temporal resolution and low cost, permitting real-time investigation of pathological processes at molecular level and sensitive detection of diseases for intraoperative imaging-guided surgery^2–5^. However, most optical techniques detect fluorescent signals generated upon real-time light excitation, wherein the background noise from endogenous molecules in biological subjects are inevitable^6^. Such a flaw challenges reliable detection of signals, giving rise to minimized signal-to-background ratio (SBR), limited penetration depth and consequently compromised imaging sensitivity^7,8^.

Real-time light-excitation-free optical agents including chemiluminescent, bioluminescent, Cerenkov, and afterglow (or persistent luminescent) probes can circumvent the interference of tissue autofluorescence^9^. However, each has its own trade-offs. For instance, chemiluminescent and bioluminescent agents utilize chemical reactions that, respectively, require reactive oxygen species and enzyme to catalyze the decomposition of substrates to trigger luminescence^10,11^, and their imaging sensitivity is usually perturbed by cellular environment and substrate availability^12,13^. On the contrary, Cerenkov and afterglow agents do not require particular chemical mediator or exogenous enzyme, and thus have higher versatility for imaging applications. However, Cerenkov agents are basically radioisotopes and intrinsically limited to only emit visible light^14,15^, thus their biomedical applications are challenged by both the biosafety issue of radiotracers and shallow imaging depth due to short-wavelength emission. Differently, afterglow agents act as the optical battery to trap irradiated photoenergy in defects and then slowly release the stored energy by photonic emission upon physical (thermal, mechanical, etc.) activation, eliminating the need of invasive radiotracers or exogenous mediators^16^.

Despite the advantage of afterglow imaging over other excitation-free strategies^17^, there are only two kinds of afterglow nanoagents, which are the rare-earth metal (e.g., Europium, Praseodymium) containing inorganic nanoparticles and poly[2-methoxy-5-(2-ethylhexyloxy)-1,4-phenylenevinylene] (MEHPPV)-based organic nanoparticles^18–23^. The inorganic agents are limited to the general formulas (e.g., ZnGa_2(1−x)_Cr_2x_O_4_), which may suffer from potential leakage of heavy-metal ions^24,25^. In contrast, the MEHPPV based nanoparticles have demonstrated high biocompatibility in living mice. Because of the higher SBR of afterglow relative to fluorescence (up to ~150-fold)^22^, these afterglow agents have been utilized for in vivo cell tracking^26,27^, monitoring of biomarker (e.g., glutathione, ascorbic acid)^28,29^, visualization of vascularization^24^, lymph node mapping^30^, monitoring of drug-induced hepatoxicity^22^, in vivo temperature indication^31^, and cancer theranostics^32–36^. However, to fully explore the potential of afterglow imaging in fundamental biology and clinical practice, versatile afterglow agents with bright and tunable emission are of high demand.

**Fig. 1:**
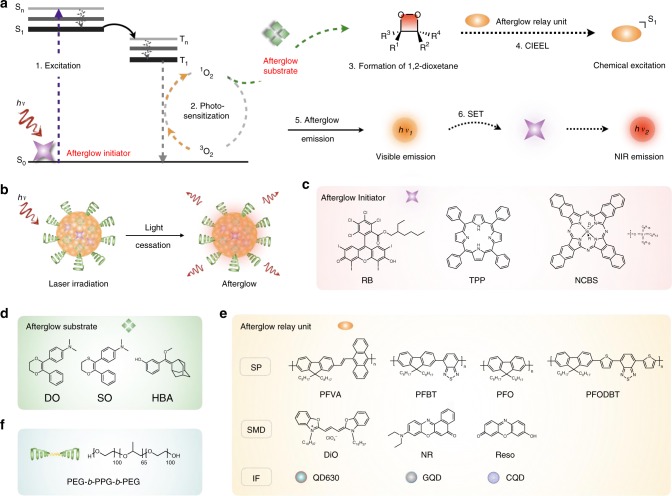
The design approach toward afterglow luminescent nanoparticles (ALNPs). **a** Schematic illustration of detailed intraparticle photoreaction processes leading to afterglow. **b** Illustration of afterglow imaging of ALNPs. **c**–**e** Chemical structures of afterglow initiators (**c**), afterglow substrates (**d**), afterglow relay units (**e**), and amphiphilic copolymer (**f**). PFVA, poly[(9,9′-dioctyl-2,7-divinylenefluorenylene)-*alt*-(9,10-anthracene)]; PFBT, poly[(9,9′-dioctylfluorenyl-2,7-diyl)-*alt*-(benzo[2,1,3]thiadiazol-4,7-diyl)]; PFO, poly(9,9′-dioctylfluorenyl-2,7-diyl); PFODBT, poly[2,7-(9,9′-dioctylfluorene)-*alt*-4,7-bis(thiophen-2-yl)benzo-2,1,3-thiadiazole]; DiO, 3,3′-dioctadecyloxacarbocyanine perchlorate; NR, nile red; Reso, resorufin; QD630, CdSe/ZnS core-shell type quantum dot

Herein, we report a generic approach to transform ordinary fluorescent agents into afterglow luminescent nanoparticles (ALNPs) for in vivo imaging. This approach relies on an intraparticle cascade photoreaction of three key components termed as afterglow initiator, afterglow substrate, and afterglow relay unit (Fig. 1a) to store the photoenergy as the chemical defects for delayed luminescence after cessation of light excitation. Within ALNPs, a photosensitizer serves as the afterglow initiator to absorb and convert photoenergy into signaling singlet oxygen (^1^O_2_); a ^1^O_2_-reactive molecule serves as the afterglow substrate to absorb and react with ^1^O_2_, forming the unstable chemiluminescent intermediate (1,2-dioxetane); and a fluorescent agent behaves as the afterglow relay unit to accept the energy from 1,2-dioxetane via chemically initiated electron exchange luminescence (CIEEL), gradually releasing it in the form of photons. Depending on whether there is an efficient secondary energy transfer (SET) between the fluorescent agent and the photosensitizer, the ultimate afterglow emission spectrum could be close to that of the fluorescent agent or the photosensitizer.

## Results

### Preparation of ALNPs

To examine the generality of the afterglow design, different kinds of substances were chosen for each component to prepare the ALNPs. Three photosensitizers including RB (rose bengal octyl ester) (Supplementary Figs. 1, 2), TPP (meso-tetraphenylporphyrin), and NCBS (silicon 2,3-naphthalocyanine bis(trihexylsilyloxide)) were used as the representative afterglow initiators (Fig. 1c), which had the excitation and emission maxima at 576 and 591 nm for RB, 418 and 650 nm for TPP, and 774 and 780 nm for NCBS, respectively (Supplementary Figs. 3, 4). Thus, the afterglow luminescence could be triggered by light irradiation at different wavelengths. Three ^1^O_2_-responsive chemiluminescent molecules including DO (*N*,*N*-dimethyl-4-(3-phenyl-5,6-dihydro-1,4-dioxin-2-yl)aniline), SO (*N*,*N*-dimethyl-4-(2-phenyl-5,6-dihydro-1,4-oxathiin-3-yl)aniline), and HBA (3-((1*r*,3*r*,5*R*,7*S*)-adamantan-2-ylidene(methoxy)methyl)phenol) were chosen as the afterglow substrates (Fig. 1d, Supplementary Figs. 5–7)^37,38^, which had chemiluminescent emission ranging from 350 to 550 nm. Three kinds of commonly used fluorescent agents were tested as the afterglow relay units (Fig. 1e), which included semiconducting polymers (SPs), small-molecule dyes (SMDs), and inorganic fluorophores (IFs), such as semiconductor quantum dots (QDs), graphene quantum dots (GQDs), and carbon quantum dots (CQDs). The fluorescence emissions of these agents ranged from visible to NIR region, providing the feasibility to fine-tune the afterglow profiles of ALNPs.

**Fig. 2:**
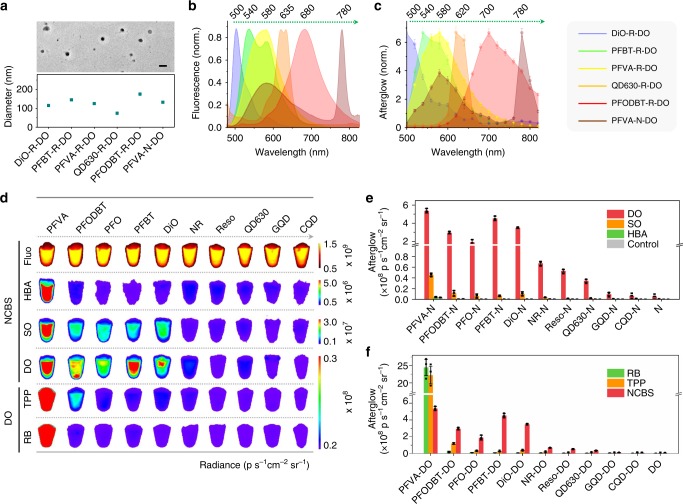
Characterization of ALNPs with different components. **a** Representative TEM image of PFVA-N-DO ALNPs (upper panel) and representative hydrodynamic diameters of various ALNPs measured by DLS (bottom panel) in 1 × PBS (phosphate buffered saline) buffer (pH = 7.4). Scale bar: 200 nm. **b** Normalized (norm.) fluorescence spectra of representative ALNPs with the emission ranging from visible to NIR region in 1 × PBS buffer (pH = 7.4). **c** Normalized afterglow luminescence spectra of ALNPs in (**b**) in 1 × PBS buffer (pH = 7.4). **d** Fluorescence and afterglow images of ALNPs with various components. Fluorescence images of NCBS based ALNPs were acquired at 780 nm upon excitation at 710 nm. Afterglow images were captured after light pre-irradiation (1 W cm^−2^ 808 nm for NCBS based ALNPs while 0.1 W cm^−2^ white light for RB or TPP based ALNPs) for 5 s. For all ALNPs, [afterglow initiator] = 0.75 μg mL^−1^ (2.5 w/w%), [afterglow substrate] = 15 μg mL^−1^ (50 w/w%), [afterglow relay unit] = 30 μg mL^−1^. **e** Quantification of afterglow intensities of ALNPs with the same afterglow initiator (NCBS) whereas varying substrates and relay units in (**d**). **f** Quantification of afterglow intensities of ALNPs with the same afterglow substrates (DO) whereas varying afterglow initiators and relay units in (**d**). Error bars indicated standard deviations of three separate measurements

The afterglow contrast agents were prepared with different combinations of afterglow initiator, substrate, and relay unit through co-nanoprecipitation with an amphiphilic copolymer PEG-*b*-PPG-*b*-PEG (Fig. 1f, Supplementary Fig. 8). The doping ratios for each component within the ALNPs were optimized (Supplementary Figs. 9–10). The solutions of resulted 50 kinds of ALNPs were translucent with no obvious precipitates after preparation (Supplementary Figs. 11&12). Dynamic light scattering (DLS) revealed the hydrodynamic diameters of the ALNPs ranged from 80 to 180 nm (Fig. 2a, Supplementary Fig. 11b, c), except for CQD-based ALNPs (12 nm). This should be attributed to the intrinsic hydrophilicity and surface charge of CQD. Furthermore, transmission electron microscope (TEM) revealed the spherical morphology of these ALNPs (Fig. 2a).

Afterglow luminescence of these ALNPs were recorded with optimized light irradiation time (5 s) (Supplementary Fig. 13). Because of different absorption (Supplementary Fig. 14), NCBS-doped ALNPs were irradiated with 808 nm laser (1 W cm^−2^) while RB- or TPP-doped ALNPs were irradiated with white light (0.1 W cm^−2^). As expected, luminescence was detected from ALNPs after cessation of laser irradiation (Fig. 2d), which was barely detectable for the nanoparticles consisting of only afterglow initiator and substrate or relay unit (Fig. 2e, f). This validated the proposed afterglow mechanism and the collaborative roles of three components. Dependent on the compositions of ALNPs, the afterglow luminescence spectra ranged from visible to NIR region (Fig. 2b, c). In general, the afterglow spectra of ALNPs were similar to the corresponding fluorescence spectra (Supplementary Figs. 15–17). If there was energy transfer from afterglow relay unit to initiator, the shape of afterglow spectrum could be more like that of the initiator. Otherwise, the afterglow emission was closer to that of the relay unit. For instance, ALNPs consisting of PFO, NCBS, and DO (termed as PFO-N-DO) had a strong NIR afterglow emission at 780 nm because of the secondary energy transfer from PFO to NCBS; whereas ALNPs consisting of PFO, RB, and DO (termed as PFO-R-DO) only had the afterglow emission from PFO due to the inefficient energy transfer from PFO to RB. Discrepancy in the fluorescence and afterglow spectral profiles was observed for several ALNPs (especially TPP-doped ones) such as GQD-N-DO, DiO-TPP-DO (termed as DiO-T-DO), CQD-R-DO, etc. This should be ascribed to the fact that the afterglow photophysical process was different from that of fluorescence (Supplementary Fig. 18): in the fluorescence process, light excitation only led to the emission of the fluorescent agent, which was followed by the potential energy transfer to the photosensitizer; whereas, in the afterglow process, in addition to such a potential energy transfer, the photosensitizer could be directly excited through the energy released from the high-energy intermediate (1,2-dioxetane). Thus, the photophysical interplay between the afterglow initiator and the relay unit offered additional space to fine-tune the afterglow profiles of ALNPs, potentially enabling multiplexed imaging (Supplementary Fig. 19).

The afterglow intensities of ALNPs were different from each other (Fig. 2d–f). Comparison of the nanoparticles with the same afterglow initiator (NCBS) and relay unit revealed that DO-doped nanoparticles had the brightest afterglow luminescence among three afterglow substrates (Fig. 2e), which was followed by SO and HBA doped ones. Moreover, among all the tested fluorescence agents, PFVA-based nanoparticles had the highest afterglow intensities provided that other two components were the same. For instance, PFVA-N-DO gave the brightest afterglow luminescence among all NCBS-doped ALNPs, which was 12- and 123-fold higher than that of PFVA-N-SO, and PFVA-N-HBA nanoparticles, respectively. Variation of photosensitizer also impacted the afterglow luminescence of ALNPs, because the ability to generate ^1^O_2_ was different. NCBS-doped ALNPs generally had brighter afterglow luminescence than TPP- and RB-doped ones when other components were the same (Fig. 2f). However, this was not the case when the fluorescent agent was PFVA. For instance, afterglow intensities of PFVA-R-DO and PFVA-T-DO were 4.6- and 4.1-fold higher than that of PFVA-N-DO, respectively. This was because of the additional amount of ^1^O_2_ generated by PFVA under white light irradiation but not 808 nm irradiation. Thus, the afterglow intensities of ALNPs were determined by all three components, but independent of particle size if the components were the same (Supplementary Fig. 20). Moreover, the afterglow could be repeatedly induced (Supplementary Fig. 21), or restored after preservation of pre-irradiated ALNPs at −20 °C (Supplementary Fig. 22).

### Quantitative analysis and prediction of afterglow intensity

**Fig. 3:**
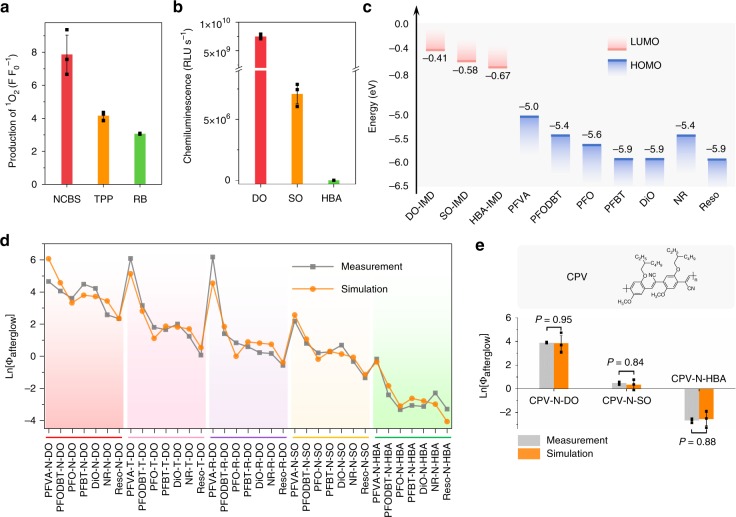
Mechanistic study of descriptors in the estimation of afterglow intensity of ALNPs. **a** Quantification of ^1^O_2_ generating capability for the different afterglow initiator doped nanoparticles at identical mass concentration (0.75 μg mL^−1^) in 1 × PBS (pH = 7.4). NCBS nanoparticles were pre-irradiated at 808 nm for 5 s (at 1 W cm^−2^), while TPP and RB nanoparticles were pre-irradiated with white light for 5 s (0.1 W cm^−2^). Production of ^1^O_2_ was defined as the fluorescence enhancement (*F* *F*_0_^−1^) of ^1^O_2_ sensor green (SOSG, 1 μM) at 524 nm. **b** Chemiluminescent intensities (RLU s^−1^) of DO, SO, and HBA in the presence of NCBS in tetrahydrofuran after pre-irradiation at 808 nm for 5 s (at 1 W cm^−2^). [DO] = [SO] = [HBA] = 15 μg mL^−1^; [NCBS] = 0.75 μg mL^−1^. RLU, relative light unit. **c** Schematic illustration of energy levels of afterglow substrates and afterglow relay units^39,47,48^. LUMO, lowest unoccupied molecular orbitals. DO/SO/HBA-IMD, DO/SO/HBA-based dioxetane intermediate. **d** Measured (measurement) and estimated (simulation) afterglow intensities of ALNPs. Estimated afterglow intensities were calculated from Eq. (1). **e** Prediction of afterglow intensities of a new afterglow relay unit CPV by Eq. (1). *P* values were calculated by Student’s two-sided *t*-test. Inset: chemical structure of CPV. Error bars indicated standard deviations of three separate measurements

To quantitatively analyze the factors governing the afterglow intensity of ALNPs, a mathematic model was proposed. Relative afterglow intensity (Φ_Afterglow_) was defined as the ratio of the afterglow luminescence of individual ALNP to that of the control ALNP which consisted of NCBS and DO without afterglow relay units (termed as N-DO). Based on the detailed afterglow process (Supplementary Fig. 23), four descriptors were retrieved and defined for simulation. First of all, because afterglow process started from photosensitization, the production of ^1^O_2_ by afterglow initiator (Φs_1_) was defined as the first descriptor (Fig. 3a). As previously reported^39,40^, chemiluminescent property of afterglow substrate and the oxidation potential of fluorescent agent (afterglow relay unit) are important to CIEEL. Therefore, the chemiluminescent quantum yield of afterglow substrate after reaction with ^1^O_2_ (Φ_Cl_) was selected as the second descriptor (Fig. 3b); moreover, corresponding to oxidation potential, the energy level of highest occupied molecular orbital (HOMO) of afterglow relay unit (*E*_HOMO_) with respect to frontier molecular orbital theory (Fig. 3c, Supplementary Fig. 24) was defined as the third descriptor. Because the afterglow emission in this generic approach involved the potential SET between the afterglow relay unit and the afterglow initiator, the relative fluorescence efficiency of ALNP (η_Fl_) was defined as the last descriptor. This descriptor was determined by both initiator and relay unit and was quantified by the ratio of the integrated total fluorescence intensity of individual ALNP to that of the control ALNP N-DO (Supplementary Fig. 25). After supervised learning analysis^41,42^, a simulated equation which correlated four descriptors with Φ_Afterglow_ was generated as shown below (see Methods for calculation details):

1 $$\Phi _{{\mathrm{Afterglow}}} = \Phi {\mathrm{s}}_1^{2.76} \times \Phi _{{\mathrm{Cl}}}^{0.46} \times {\mathrm{e}}^{\left[ {2.70 \times E_{{\mathrm{HOMO}}} + 13.13} \right]} \times {\mathrm{\eta }}_{{\mathrm{Fl}}}^{0.41}$$

According to statistical results (Supplementary Fig. 26), all descriptors were strongly correlated with Φ_Afterglow_ (Supplementary Fig. 26c, *P* < 0.05). The calculated Eq. (1) showed an impressive coefficient of determination (*R*^2^) of 0.944 (adjusted *R*^2^ = 0.936), and the measured and simulated Φ_Afterglow_ values involved in quantitative analysis showed close proximity to each other (Fig. 3d), both suggesting the excellent fitness of Eq. (1) to this afterglow model. Based on Eq. (1), it was apparent that Φ_Afterglow_ demonstrated non-linear increment with four descriptors related to the cross-talk of three major afterglow components. Basically, increased production of ^1^O_2_, chemiluminescence of afterglow substrate, HOMO energy level of afterglow relay unit, or fluorescent efficiency of fluorescent units in ALNP could contribute to brightening afterglow. Such a pattern corresponded well with the experimental data in Fig. 2. Thereby, these statistical data demonstrated the rationality of descriptor selection for quantitative analysis and implied the feasibility of Eq. (1) for afterglow prediction.

To test the predictive capability of the proposed equation, another fluorescent agent, CPV, was used as the afterglow relay unit. Note that CPV hardly emitted afterglow luminescence by itself^22^. However, after the nanoformulation through doping CPV with afterglow initiator (NCBS) and substrate (DO, SO, or HBA), intense afterglow luminescence was detected (Supplementary Fig. 27). The comparison of experimentally measured and Eq. (1) simulated Φ_Afterglow_ of CPV-based ALNPs was shown in Fig. 3e. Impressively, no significant difference was observed between the measured and estimated Ln[Φ_Afterglow_] (*P* > 0.05), validating the prediction reliability of Eq. (1) to estimate afterglow luminescence for this generic afterglow approach.

### Tissue penetration of afterglow luminescence

**Fig. 4:**
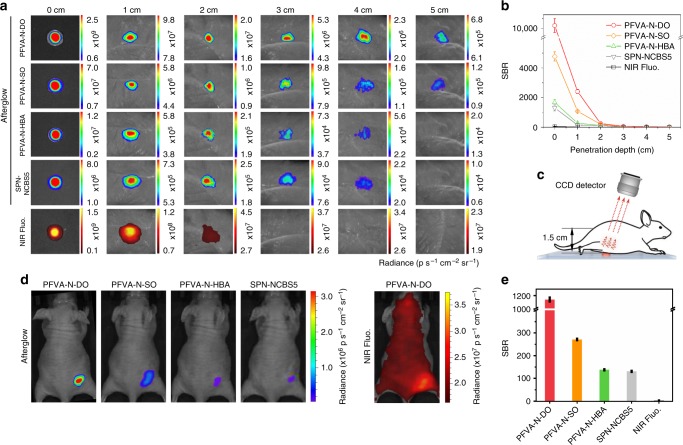
Tissue penetration study of ALNPs. **a** Afterglow and NIR fluorescence images of PFVA-based ALNPs ([PFVA] = 100 μg mL^−1^, [DO], [SO], or [HBA] = 50 μg mL^−1^, [NCBS] = 2.5 μg mL^−1^, 50 μL) and SPN-NCBS5 nanoparticles ([MEHPPV] = 100 μg mL^−1^, [NCBS] = 5 μg mL^−1^, 50 μL) under different depths of chicken breast tissues. **b** SBRs of afterglow or fluorescence images in (**a**) at different tissue depths. **c** Schematic illustration of optical imaging through a living mouse. Afterglow and NIR fluorescence images of PFVA-based ALNPs or SPN-NCBS5 were captured after penetrating through a living mouse. CCD, charged coupled device. **d** Afterglow and fluorescence images of PFVA-based ALNPs or SPN-NCBS5 nanoparticles penetrating through a living mouse. Concentrations of nanoparticles were identical to those in (**b**). **e** Quantification of the SBRs of afterglow or fluorescence images in (**d**). Both the PFVA-based ALNPs and SPN-NCBS5 were pre-irradiated at 808 nm for 5 s (at 1 W cm^−2^) before imaging. Fluorescence images were captured at 780 nm upon excitation at 710 nm. Error bars indicated standard deviations of three separate measurements

To assess the imaging capability of ALNPs, we examined the imaging performance of ALNPs in comparison with NIR fluorescence at different tissue depths. Moreover, the afterglow performance was benchmarked against the reported afterglow agent SPN-NCBS5. SPN-NCBS5 was similarly prepared via nanoprecipitation, wherein MEHPPV was doped with 5 w/w% NCBS using PEG-*b*-PPG-*b*-PEG as the matrix^22^. Considering strong afterglow luminescence and NIR emission at 780 nm, PFVA-N series with long-term stability and good cytocompatibility were selected as the representative ALNPs (Supplementary Figs. 28–30). With the increase of tissue depth, both NIR fluorescence and afterglow luminescence intensities from the buried ALNPs significantly decreased (Fig. 4a). Because of the minimized background noise of afterglow imaging, the SBRs of afterglow images were remarkably higher than those of NIR fluorescence images at all tissue depths (Fig. 4b). Notably, the NIR fluorescence was almost undetectable at 3 cm (SBR close to 1), whereas the afterglow luminescence could still be clearly visualized (SBR: 61 ± 8; SBR is expressed as mean ± standard deviation of three independent measurements). These data suggested the superior imaging performance of afterglow luminescence over NIR fluorescence. Moreover, depending on afterglow substrate, PFVA-N ALNPs showed similar (PFVA-N-HBA) or even higher (PFVA-N-DO/SO) afterglow SBRs than SPN-NCBS5 at the same tissue depth. For instance, at a tissue depth of 2 cm, PFVA-N-HBA showed similar SBR (122 ± 1) to SPN-NCBS5 (116 ± 1), whereas the SBRs of PFVA-N-DO (248 ± 10) and SO (191 ± 36) were, respectively, 2.1 and 1.6-fold higher than that of SPN-NCBS5, mainly attributed to the much higher afterglow brightness (Fig. 4a). In particular, PFVA-N-DO (or SO) reached a maximum imaging depth of 5 cm (SBR: 26 ± 1 for PFVA-N-DO and 6 ± 1 for PFVA-N-SO), which was deeper than 4 cm by SPN-NCBS5 (SBR: 3 ± 1). These results not only indicated the advantage of PFVA-N ALNPs over SPN-NCBS5 for afterglow imaging, but also emphasized the design flexibility of ALNPs to further promote penetration depth and imaging sensitivity.

The deep-tissue imaging capability of PFVA-N ALNPs was further validated in living animals. As illustrated in Fig. 4c, nanoparticle solutions were placed beneath a living mouse wherein the tissue depth was measured to be 1.5 cm. Because of the strongest afterglow intensity and low background noise (Fig. 4d), the afterglow of PFVA-N-DO had the highest SBR (1136 ± 19), followed by PFVA-N-SO (272 ± 3) and PFVA-N-HBA (138 ± 1) (Fig. 4e). Remarkably, the afterglow SBR of PFVA-N-DO exceeded that of SPN-NCBS5 (131 ± 3) by 8.7 times. On the other hand, the NIR fluorescence from PFVA-N-DO could hardly be differentiated from the background (tissue autofluorescence). These data thus corresponded well with in vitro tissue penetration study, validating the ability of ALNPs for ultrasensitive deep-issue afterglow imaging.

### In vivo tumor imaging and biodegradation study

**Fig. 5:**
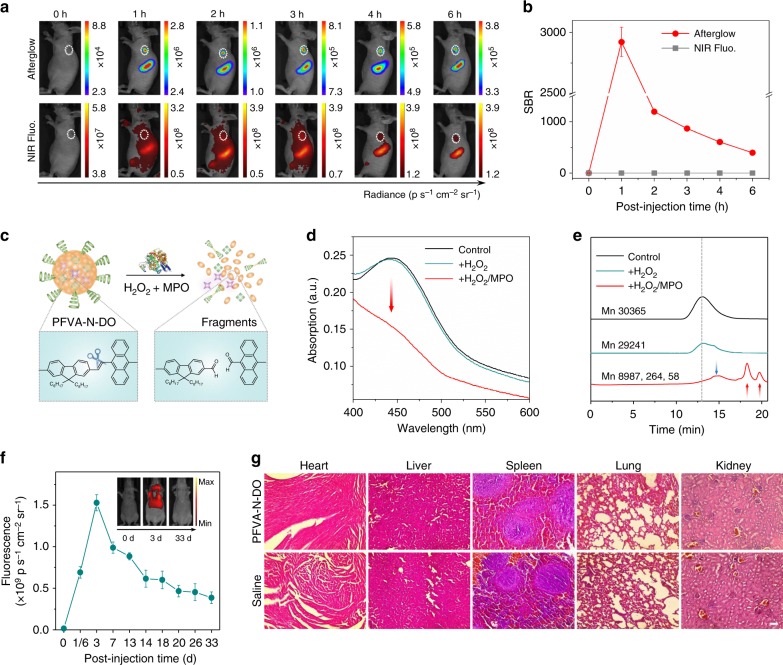
In vivo imaging, biodegradation, and clearance studies of ALNPs. **a** Representative afterglow and NIR fluorescence images of 4T1 xenograft tumor bearing mice at the different time points after tail vein injection of PFVA-N-DO ([PFVA] = 250 μg mL^−1^, [DO] = 125 μg mL^−1^, [NCBS] = 6.25 μg mL^−1^, 250 μL). Afterglow images were acquired after pre-irradiation of mice at 808 nm laser for 5 s (0.3 W cm^−2^). Fluorescence images were acquired at 780 nm upon excitation at 710 nm. White dashed circles indicated location of tumor. **b** SBRs of afterglow and NIR fluorescence imaging in tumor region as a function of time in (**a**). **c** Proposed mechanism of biodegradation of PFVA-N-DO nanoparticles by the mixture of MPO and H_2_O_2_. **d** Absorption spectra of PFVA-N-DO ([PFVA] = 4 μg mL^−1^) after incubation with buffer (control), H_2_O_2_ (300 mM), or MPO (50 μg mL^−1^)/H_2_O_2_ (300 mM) at 37 °C for 24 h in 100 mM PBS (pH = 7.0). **e** GPC results of ALNP solutions in (**d**). **f** Quantification of the NIR fluorescence intensities of liver region in living mice as a function of time after intravenous injection of PFVA-N-DO ([PFVA] = 250 μg mL^−1^, [DO] = 125 μg mL^−1^, [NCBS] = 6.25 μg mL^−1^, 250 μL). NIR fluorescence images were acquired at 780 nm upon excitation at 710 nm. **g** Hematoxylin & eosin staining of major organs from mice after tail vein injection of PFVA-N-DO ALNPs ([PFVA] = 250 μg mL^−1^, [DO] = 125 μg mL^−1^, [NCBS] = 6.25 μg mL^−1^, 250 μL) or saline (250 μL) for 33 days. Scale bar: 50 µm. Error bars indicated standard deviations of three separate measurements (*n* = 3)

To evaluate afterglow performance of ALNPs for in vivo imaging, PFVA-N-DO was selected as the representative ALNP to image tumor in living mice in comparison with NIR fluorescence. After systemic administration of PFVA-N-DO, the afterglow SBR in tumor region dramatically increased, leading to the visualization of tumor at 1 h post injection (Fig. 5a). In contrast, the NIR fluorescence SBR slightly increased (Fig. 5b), and thus the tumor was only detectable at 4 h post injection. Note that at 1 h post injection, the afterglow SBR in tumor region (2922 ± 121) was three orders of magnitude higher than that of NIR fluorescence (~1). Such a afterglow SBR was not only higher than fluorescence imaging in both first and second NIR window (SBRs up to ~135)^43^, but also significantly exceeded the SBRs of other excitation-free imaging modalities including chemiluminescence (up to ~20)^44^, bioluminescence (up to ~1000)^10^, and Cerenkov luminescence imaging (up to ~154) (Supplementary Table 1)^45^. This should be mainly attributed to the fact that chemiluminescence, bioluminescence, and Cerenkov luminescence imaging usually rely on visible emission. Ex vivo data revealed that the uptake of PFVA-N-DO in tumor was 0.58-fold of that in liver (Supplementary Fig. 31), further confirming its ability to passively target tumor. These data highlighted that by PFVA-N-DO mediated afterglow imaging allowed for rapider detection of tumor with the superior contrast and sensitivity over other optical agents.

Biodegradation and in vivo clearance study were subsequently performed to examine the biosafety of PFVA-DO-N. To mimic in vivo environment, myeloperoxidase (MPO) abundantly expressed in phagocytes was used as the oxidative enzyme for in vitro biodegradation (Fig. 5c). In the presence of hydrogen peroxide (H_2_O_2_), MPO catalyzes the production of hypochlorous acid (HClO) to digest foreign substances^46^. After overnight incubation of PFVA-N-DO with MPO and H_2_O_2_, the absorbance at 450 nm assigned to PFVA significantly dropped (Fig. 5d), suggesting the fragmentation of PFVA by MPO. The biodegradation was further confirmed by gel permeation chromatography (GPC), as indicated by the evidently decreased molecular weight of PFVA after MPO treatment (Fig. 5e). Such an efficient degradation should be ascribed to the oxidation induced cleavage of double bonds in the conjugated backbones of PFVA (Fig. 5c), which was previously reported^22,30^.

To monitor the in vivo clearance of PFVA-N-DO, they were systemically administered into mice followed by long-term NIR fluorescence recording (Fig. 5f). After administration, NIR fluorescent signals from liver increased over time and reached the maximum at 3 days post injection. Later, the NIR fluorescence from liver continuously decreased to almost undetectable level at 33 days post injection (Supplementary Fig. 32). These results indicated the long-term clearance of PFVA-N-DO via hepatobiliary excretion in living animals. Furthermore, no noticeable histological damage was observed in the major organs of living mice after systemic administration of PFVA-N-DO for 33 days (Fig. 5g), suggesting the good biocompatibility of PFVA-N-DO.

## Discussion

In summary, we reported a generic approach that transformed traditional fluorescent agents into a new library of afterglow agents (ALNPs). By virtue of an efficient intraparticle cascade photoreaction of three key components (afterglow initiator, substrate, and relay unit), ALNPs were able to chemically store the photoenergy and spontaneously emit long-lived luminescence after cessation of optical excitation. Such a facile approach was applicable to a wide range of compositions: RB, TPP, or NCBS for the initiator, DO, SO, or HBA for the substance, and inorganic or organic fluorophores for the relay units. The sophisticated but fairly controllable photochemical interactions within the nanoparticles allowed to fine-tune the afterglow emission from visible to NIR region by adjusting the ALNP compositions. To elucidate the factors involved in this generic afterglow process, a 4-descriptor based mathematical model (Eq. (1)) was generated from supervised learning analysis, which accurately predicted the afterglow intensities of unknown ALNP composition. Using PFVA-N-DO ALNPs as an example, the afterglow achieved a maximal imaging depth at 5 cm in biological tissue, deeper than the reported afterglow agents (4 cm). As compared with NIR fluorescence, the afterglow of ALNPs exhibited three orders of magnitude higher SBR (2922 ± 121), allowing for rapider detection of tumor in living mice after systematic administration. To the best of our knowledge, this is the highest SBR achieved so far for in vivo optical imaging regardless of their optical modalities and detection wavelengths. In conjunction with the heavy-metal-free benign nature, the representative PFVA-N-DO ALNPs were enzymatically biodegradable and clearable with a good long-term biocompatibility, further ensuring their in vivo applications. Thus, our study showed a controllable nanoengineering approach nearly applicable to all kinds of fluorophores regardless of their composition for background-free molecular optical imaging.

## Methods

### Chemicals and characterization

All materials were purchased from Sigma-Aldrich Pte. Ltd. unless otherwise noted. PFVA, PFO, and PFBT were purchased from Luminescence Technology Corp. DO and SO were purchased from Aberjona Laboratories, Inc.

DLS profiles of nanoparticles were measured by Malvern Nano-ZS Particle Sizer. TEM images of nanoparticles were captured by JEOL JEM 1400 TEM with an acceleration rate of 100 kV. Proton nuclear magnetic resonance (^1^H NMR) spectra were measured by Bruker Avance 300 MHz NMR. Electrospray ionization mass spectrometry (ESI-MS) spectrum of RB was measured by ThermoFinnigan LCQ Fleet MS equipped with Themo Accela LC and ESI source. Absorption spectra were measured on a Shimadzu UV-2450 spectrophotometer. Fluorescence spectra and fluorescence efficiency were acquired on a Fluorolog 3 spectrofluorometer (HORIBA, Ltd.). Chemiluminescence of afterglow substrates was recorded with a Luminometer (Promega, USA). Molecular weights of PFVA ALNPs in biodegradation studies were characterized by GPC using THF as the eluent and polystyrene as the standard. White light source for afterglow luminescence was supplied by an LED Fiber Optic Illuminator (L-150A) with an output power density of 0.1 W cm^−2^ (wavelength range: 400–800 nm). Fiber coupled 808 nm laser system was purchased from Changchun New Industries Optoelectronics Tech. Co., Ltd. NIR fluorescence and afterglow images were acquired by IVIS SpectrumCT In Vivo Imaging System (PerkinElmer, Inc.).

### Synthesis of RB

Compound 1 (1.0 g; 1.0 mmol) and 2-ethylhexyl bromide (0.5 g; 2.6 mmol) were dissolved and magnetically stirred in *N*,*N*-dimethylformamide (DMF) at 80 ℃. After 6 h, excess 2-ethylhexyl bromide and DMF were removed by rotary evaporation. The residue was then dissolved in diethyl ether. To remove compound 6 and inorganic salts, the residue in diethyl ether was washed with water for three times followed by desiccation using anhydrous Na_2_SO_4_. The obtained residue was further purified by column chromatography using ethyl acetate as the eluent to afford the final product RB in deep purple color. ^1^H NMR (300 MHz, DMSO-*d*_6_, Supplementary Fig. 2) *δ* (ppm): *δ* = 0.78–0.83 (m, 6H), 1.24 (m, 8H), 1.45 (m, 1H), 3.86–3.88 (d, *J* = 6 Hz, 2H), 7.51–7.52 (s, 2H). ESI-MS (*m*/*z*): calculated: 1084.61; found: 1084.95.

### Synthesis of HBA

HBA was synthesized following the method in literature^37^. Briefly, synthesis of HBA was started from the commercially available 3-hydroxybenzaldehyde by protecting with trimethyl orthoformate to afford 3-(dimethoxymethyl)phenol, which was followed by additional protection of the phenol group with tert-butyldimethylsilyl chloride (TBS-Cl) to give tert-butyl (3-(dimethoxymethyl)phenoxy)dimethylsilane. This compound was reacted with trimethylphosphite to produce a phosphonate derivative, and then condensed with 2-adamantanone via the Wittig-Horner reaction to provide an enol ether precursor. At last, deprotection of the TBS group of the resulted precursor gave HBA. ^1^H NMR (300 MHz, CDCl_3_, Supplementary Fig. 4) *δ* (ppm): *δ* = 1.78–1.96 (m, 12H), 2.65 (s, 1H), 3.24 (s, 1H), 3.32 (s, 3H), 5.35 (s, 1H), 6.76–6.79 (d, *J* = 9 Hz, 1H), 6.85–6.89 (m, 2H), 7.21 (t, 1H).

### Preparation of ALNPs and SPN-NCBS5

ALNPs were prepared at the optimized doping ratios of different components. Afterglow initiator (0.0025 mg), afterglow substrate (0.05 mg), afterglow relay unit (0.1 mg), and PEG-b-PPG-b-PEG (10 mg) were dissolved in THF, respectively, and then mixed together under sonication. Then THF solvent was removed by rotary evaporation to afford a thin film. The obtained film was hydrated in distilled deionized water (2 mL) under vigorous sonication to prepare ALNPs. The resulted ALNP solutions were filtrated through 0.22 µm Millipore poly(ether sulfone) syringe driven filter to remove impurities and then concentrated by ultracentrifugation. The concentrated ALNP solutions were diluted in 1 × PBS buffer (pH 7.4) and stored in dark at 4 ℃.

SPN-NCBS5 nanoparticles were prepared following the reported method^22^. MEHPPV (0.25 mg), PEG-b-PPG-b-PEG (20 mg), and NCBS (0.0125 mg) were dissolved and mixed in THF (1 mL), followed by rapid injection into deionized water and removal of THF by a gentle N_2_ flow. The resulted SPN-NCBS5 was then purified by filtration through the abovementioned 0.22 µm filter and concentrated by ultracentrifugation.

### NIR fluorescence and afterglow imaging

NIR fluorescence images of ALNPs were acquired by IVIS SpectrumCT In Vivo Imaging System under fluorescence mode with exposure time for 0.1 s. Fluorescence signals were collected with excitation at 710 nm and emission at 780 nm. Afterglow images of ALNPs were acquired by IVIS SpectrumCT In Vivo Imaging System within 5 s after laser irradiation under bioluminescence mode with open filter (exposure time: 1 s). Afterglow spectra of ALNPs were acquired at similar conditions yet with specific emission filters. Both fluorescence and afterglow intensities were quantified by region of interest (ROI) analysis using Living Imaging 4.3 Software.

### Definition and measurement of afterglow descriptors

Afterglow descriptors were measured and calculated using N-DO ALNPs as the reference. Relative afterglow intensity (Φ_Afterglow_) was calculated as the ratio of absolute afterglow intensity of individual ALNP (*I*_ALNP_) to that of N-DO (*I*_N-DO_) under identical mass concentration and laser condition ([afterglow initiator] = 0.75 μg mL^−1^, [afterglow substrate] = 15 μg mL^−1^; laser condition: 808 nm laser at 1 W cm^−2^ for NCBS-doped ALNPs while white light at 0.1 W cm^−2^ for TPP and RB-doped ALNPs for 5 s). Equation was listed as follows (Eq. (2)):

2 $${\mathrm{\Phi }}_{{\mathrm{Afterglow}}} = \frac{{I_{{\mathrm{ALNP}}}}}{{I_{{\mathrm{N - DO}}}}}$$

Chemiluminescence quantum yields (Φ_Cl_) of SO and HBA were measured and calculated relative to that of DO (Φ_Cl_ = 0.021) referring to the reported method^38^.

Production of ^1^O_2_ by afterglow initiator (Φs_1_) (0.75 μg mL^−1^, 1 mL) was calculated as the fluorescence enhancement (*F* *F*_0_^−1^) of ^1^O_2_ sensor green (SOSG, 1 μM) at 524 nm after laser irradiation (808 nm at 1 W cm^−2^ for NCBS while white light at 0.1 W cm^−2^ for TPP and RB) for 5 s (Eq. (3)).

3 $${\mathrm{\Phi s}}_1 = \frac{F}{{F_0}}$$

Fluorescence efficiency of ALNPs (η_Fl_) was calculated as the ratio of integrated fluorescence intensity of particular ALNPs $\left( {\mathop {\smallint }\limits_{500}^{840} Fl_{{\mathrm{ALNP}}}{\mathrm{d}}\lambda } \right)$ to that of N-DO $\left( {\mathop {\smallint }\limits_{500}^{840} Fl_{\mathrm{N}}{\mathrm{d}}\lambda } \right)$ with identical mass concentration ([afterglow relay unit] = 5 μg mL^−1^, [afterglow initiator] = 0.125 μg mL^−1^; excitation: 450 nm, emission: 500–840 nm) (Eq. (4)). Afterglow substrates were excluded because of their negligible influence on fluorescent emission.

4 $${\mathrm{\eta }}_{{\mathrm{Fl}}} = \frac{{\mathop {\smallint }\nolimits_{500}^{840} Fl_{{\mathrm{ALNP}}}{\mathrm{d}}\lambda }}{{\mathop {\smallint }\nolimits_{500}^{840} Fl_{\mathrm{N}}{\mathrm{d}}\lambda }}$$

HOMO energy levels of afterglow relay units were collected from either references or computational calculation.

### Calculation of energy levels

HOMO and LUMO energy levels of Reso and high-energy intermediates of DO, SO, and HBA were calculated by Gaussian 09 software based on density functional theory (DFT) with B3LYP/6-31 G(d) method.

### Cell culture and cytotoxicity assay

4T1 murine mammary carcinoma cells were purchased from ATCC (American Type Culture Collection). These cells were cultured in DMEM (Dulbecco’s Modified Eagle Medium) supplemented with 10% FBS (fetal bovine serum) and 1% antibiotics (10 U/mL penicillin and 10 mg/mL streptomycin). Flasks seeded with 4T1 cells were placed in an incubator with 5% CO_2_ and 95% O_2_ humidified air atmosphere at 37 ℃.

To examine the cytotoxicity of ALNPs, 4T1 cells were seeded in 96-well plates (6000 cells in 200 µL supplemented DMEM per well). After culture for 24 h, PFVA-N, PFVA-N-DO, PFVA-N-SO, and PFVA-N-HBA (final concentration [PFVA] = 5, 10, 30, 50 µg mL^−1^) were added to cell culture medium, respectively. After incubation of nanoparticles with cells for 24 h, the culture medium was removed, and cells were gently washed with fresh sterile 1 × PBS buffer for three times. Fresh supplemented DMEM (100 µL per well) mixed with MTS (0.1 mg mL^−1^, 20 µL per well) was then added to cells. After 3 h incubation, absorbance of culture medium at 490 nm was recorded by SpectraMax M5 microplate/cuvette reader. Because absorbance at 490 nm is proportional to the quantity of living cells, cell viability was calculated as the ratio of absorbance of sample treated cells to that of control cells.

### In vitro and in vivo tissue penetration study

PFVA-N-DO, PFVA-N-SO, PFVA-N-HBA ([PFVA] = 100 μg mL^−1^, [DO] = 50 μg mL^−1^, [NCBS] = 2.5 μg mL^−1^, 50 μL), and SPN-NCBS5 solutions ([MEHPPV] = 100 μg mL^−1^, [NCBS] = 5 μg mL^−1^, 50 μL) were placed under different depths of chicken breast tissue (0, 1, 2, 3, 4, or 5 cm). NIR fluorescence images of PFVA-N-DO beneath different depths of tissue were captured (exposure time: 0.1 s) with excitation and emission wavelength at 710 and 780 nm, respectively. Before capturing afterglow images, PFVA-N-DO, PFVA-N-SO, PFVA-N-HBA, and SPN-NCBS5 solutions were pre-irradiated with 808 nm laser at 1 W cm^−2^ for 5 s. Afterglow luminescence was then recorded under bioluminescence mode with open filter in IVIS SpectrumCT In Vivo Imaging System (exposure time: 1 s). As for in vivo tissue penetration study, nanoparticle solutions were placed under a living mouse with a tissue depth of 1.5 cm, and other procedures was the same as in vitro tissue penetration study.

Signal-to-background ratio (SBR) is calculated as the ratio of luminescence (fluorescence or afterglow) in region of interest with ALNPs to that of tissue background without ALNPs.

### Tumor mouse model

Animal experiments were carried out under the guidelines of Institutional Animal Care and Use Committee (IACUC), Sing Health. To establish tumor model, 2 million 4T1 cells suspended in supplemented DMEM were subcutaneously injected to the left shoulder of female NCr nude mouse (~6-weeks old). Tumors were allowed to grow until 7~10 mm^3^ before in vivo NIR fluorescence and afterglow luminescence imaging.

### In vivo tumor imaging

NIR fluorescence and afterglow luminescence imaging of 4T1-tumor bearing mice were carried out using IVIS SpectrumCT In Vivo Imaging System. NIR fluorescence and afterglow images were at first captured before injection of ALNPs. NIR fluorescence was acquired with excitation at 710 nm and emission at 780 nm (exposure time: 0.1 s). Afterglow images were acquired (exposure time: 1 s) after 808 nm laser irradiation of tumor region at 0.3 W cm^−2^ for 5 s. 4T1-tumor bearing NCr nude mice were then intravenously injected with PFVA-N-DO nanoparticles ([PFVA] = 250 μg mL^−1^, [DO] = 125 μg mL^−1^, [NCBS] = 6.25 μg mL^−1^, 250 μL, *n* = 3). Afterglow and NIR fluorescence images of mice were longitudinally monitored at different time points (1, 2, 3, 4, and 6 h). Quantification of afterglow luminescence and NIR fluorescence were performed by ROI analysis of tumor region using Living Imaging 4.3 Software.

Signal-to-background ratio (SBR) is calculated as the ratio of luminescence (fluorescence or afterglow) in tumor region after i.v. injection of ALNPs to that of tissue background before injection.

### Biodistribution method

After 6 h post injection of PFVA-N-DO ([PFVA] = 250 μg mL^−1^, [DO] = 125 μg mL^−1^, [NCBS] = 6.25 μg mL^−1^, 250 μL, *n* = 3), mice were euthanized by CO_2_ asphyxiation. Tumors and major organs (livers, hearts, spleens, kidneys, lungs, and intestines) were collected for ex vivo NIR fluorescence imaging (exposure time: 0.1 s) with excitation and emission wavelength at 710 and 780 nm, respectively. Quantification of fluorescence signal of individual tumor/organ was performed by ROI analysis using Living Imaging 4.3 Software.

### In vivo clearance of PFVA-N-DO

6-week-old female NCr nude mice were intravenously administered with PFVA-N-DO ([PFVA] = 250 μg mL^−1^, [DO] = 125 μg mL^−1^, [NCBS] = 6.25 μg mL^−1^, 250 μL, *n* = 3). NIR fluorescence images of mice in supine position were then long-termly monitored by IVIS SpectrumCT In Vivo Imaging System (exposure time: 0.1 s) with excitation and emission wavelengths at 710 and 780 nm, respectively. Representative images were captured at *t* = 1, 1/6, 3, 7,13, 14, 18. 20, 26, 33 day after administration of PFVA-N-DO. Fluorescence intensities were quantified by ROI analysis using Living Imaging 4.3 Software.

### Histological studies

After 33 days post injection of PFVA-N-DO (([PFVA] = 250 μg mL^−1^, [DO] = 125 μg mL^−1^, [NCBS] = 6.25 μg mL^−1^, 250 μL, *n* = 3) or saline (250 μL, *n* = 3), female NCr nude mice were euthanized by CO_2_ asphyxiation and major organs (hearts, livers, spleens, lungs, and kidneys) were collected. These organs were then fixed in 4% paraformaldehyde followed by embedment in paraffin and 10-µm sectioning. H & E staining was then performed to tissue sections referring to standard procedure. Optical images were captured by Nikon ECLIPSE 80i microscope (Nikon Instruments Inc., NY, USA).

### Data analysis

Statistical analysis was performed by IBM SPSS Statistics software. Results were demonstrated as mean ± SD. Statistical significance was evaluated by two-tailed Student’s *t* test. For all tests, a *P* value smaller than 0.05 was regarded as statistically significant. NIR fluorescence and afterglow images were analyzed using Living Imaging 4.3 Software.

## Supplementary information

Supplementary Information

Reporting Summary

## Data Availability

The authors declare that all data related to this study are available in the article/and or its Supplementary Information files or from the authors upon reasonable request.
