# Supplementary material for: A generic approach towards afterglow luminescent nanoparticles for ultrasensitive in vivo imaging
Source: Nat Commun. 2019 May 2;10:2064. doi: 10.1038/s41467-019-10119-x (PMC6497674; doi:10.1038/s41467-019-10119-x)
Supplement: Supplementary file 1 — Supplementary Information [file 41467_2019_10119_MOESM1_ESM.pdf]

Supplementary Information

A Generic Approach towards Afterglow Luminescent Nanoparticles for Ultrasensitive In Vivo Imaging

*Yuyan Jiang, Jiaguo Huang, Xu Zhen, Ziling Zeng, Jingchao Li, Chen Xie, Qingqing Miao, Jie Chen, Peng Chen, and Kanyi Pu**

School of Chemical and Biomedical Engineering, Nanyang Technological University, Singapore, 637457. Correspondence and requests for materials should be addressed to K.P. (email: kypu@ntu.edu.sg)

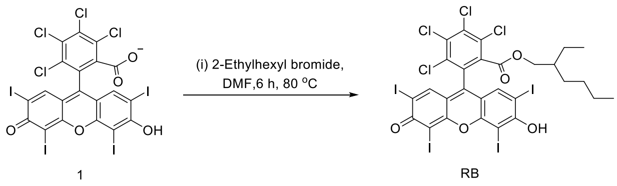

Supplementary Figure 1. Synthesis route of RB. Reagents and conditions: (i) 2-ethylhexyl bromide, DMF (dimethyl formamide), 6 h, 80 ℃.

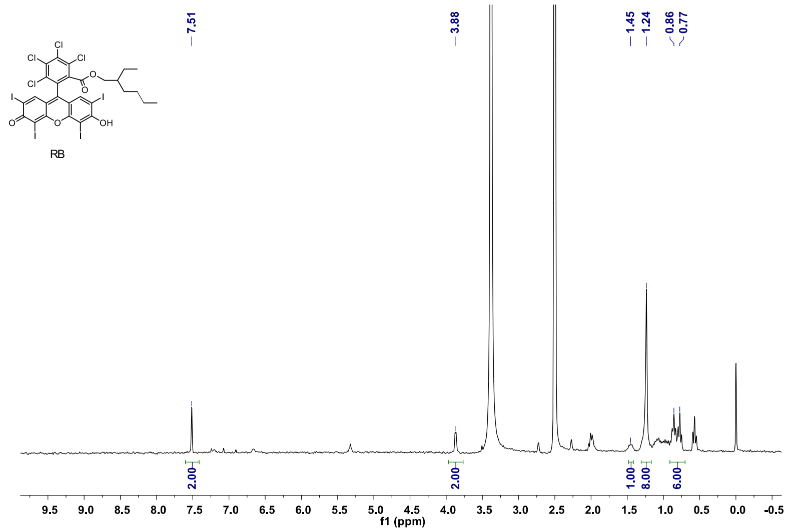

Supplementary Figure 2. ^{1}H NMR spectrum of RB.

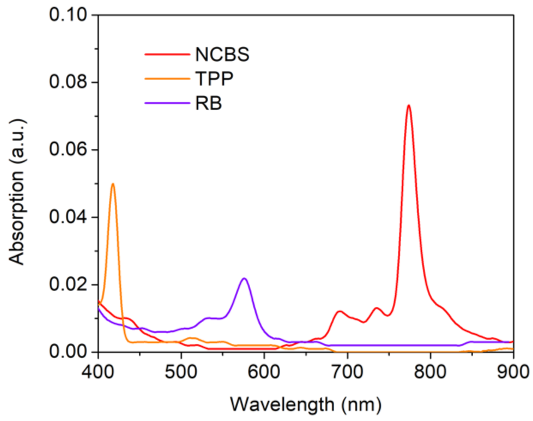

Supplementary Figure 3. Absorption spectra of NCBS, TPP and RB nanoparticles (0.125 µg mL^{-1}) in 1 × PBS buffer.

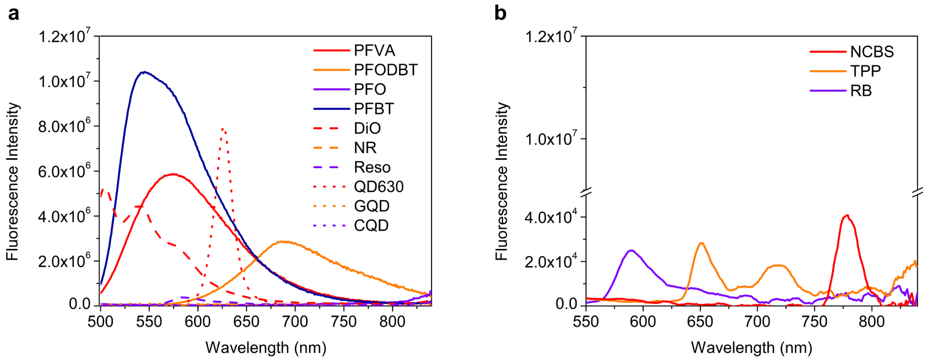

Supplementary Figure 4. Comparison of fluorescence spectra of (a) afterglow relay units (5 μg mL^{-1}) and (b) afterglow initiator (0.125 μg mL^{-1}) in nanoparticles in 1 × PBS buffer. Fluorescent excitation: 450 nm.

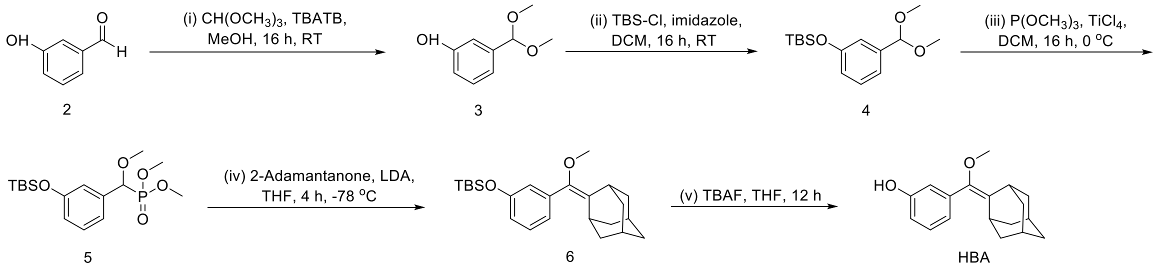

Supplementary Figure 5. Synthesis route of HBA.[1] Reagents and conditions: (i) $CH(OCH_3)_3$, TBATB (tetrabutylammonium tribromide), MeOH (methanol), 16 h, RT (room temperature); (ii) TBS-Cl (tert-butyldimethylsilyl chloride), imidazole, DCM (dichloromethane), 16 h, RT; (iii) $P(OCH_3)_3$, $TiCl_4$, DCM, 16 h, 0 °C; (iv) 2-adamantanone, LDA (lithium diisopropylamide), THF (tetrahydrofuran), 4 h, -78 °C; (v) TBAF (tetrabutylammonium fluoride), THF, 12 h.

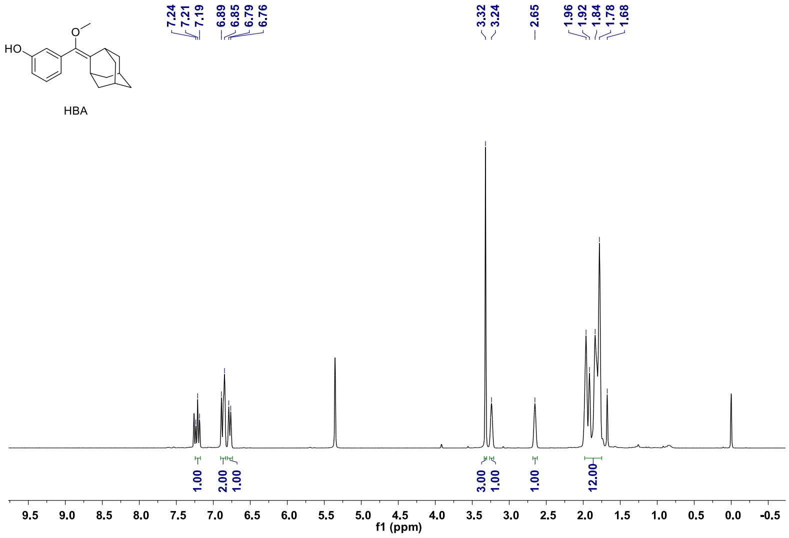

Supplementary Figure 6. ^{1}H NMR spectrum of HBA.

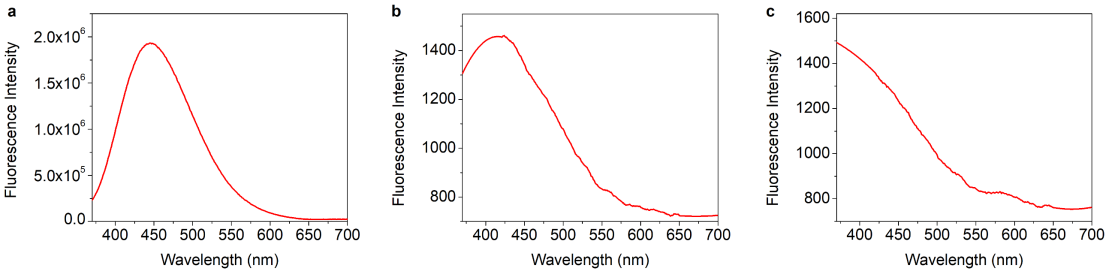

Supplementary Figure 7. Chemiluminescence spectra of (a) DO, (b) SO, and (c) HBA in THF. Chemiluminescent signal was acquired right after 808 nm (1 W cm^{-2}) laser irradiation for 5 s. [DO]=[SO]=[HBA]=25 μg mL^{-1}, [NCBS] = 1.25 μg mL^{-1}.

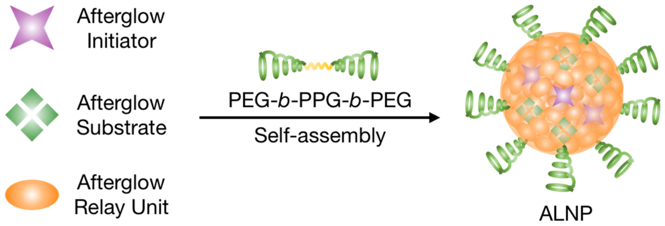

Supplementary Figure 8. Schematic illustration of preparation of ALNP.

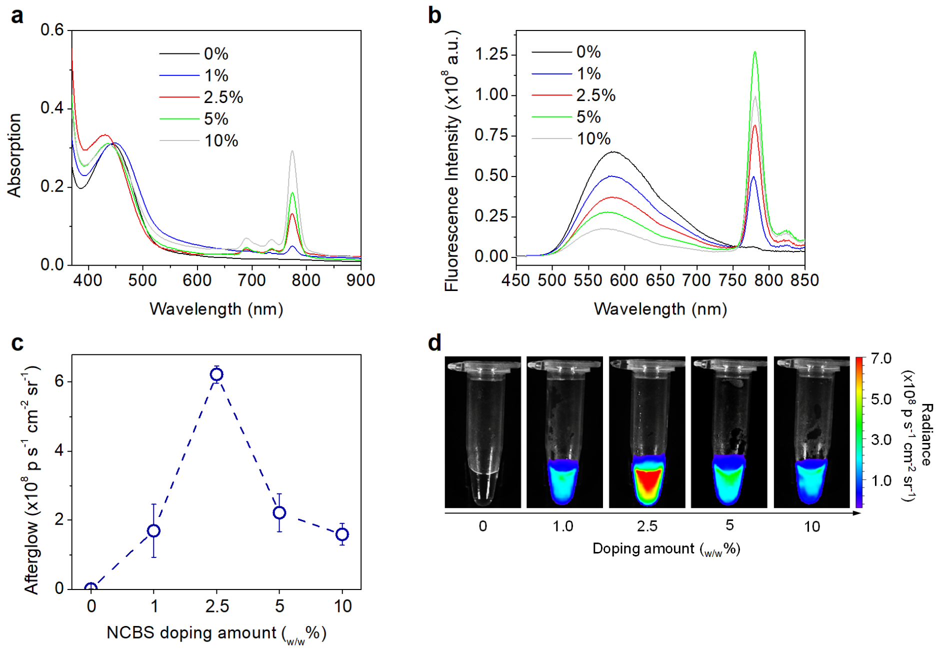

Supplementary Figure 9. Optimization of NCBS doping ratio in 1 × PBS buffer. Absorption (a) and fluorescence (b) spectra of PFVA-N-DO ALNPs with different doping amounts ($_{w/w}$%) of NCBS. [DO] = 2.5 μg mL^{-1} (50 $_{w/w}$%), [PFVA] = 5 μg mL^{-1}. (c) Afterglow intensities of PFVA-N-DO ALNPs with different doping amounts ($_{w/w}$%) of NCBS after 808 nm laser irradiation (power: 1 W cm^{-2}) for 5 s. [DO] = 15 μg mL^{-1} (50 $_{w/w}$%), [PFVA] = 30 μg mL^{-1}. (d) Afterglow images of (c) captured after laser irradiation. Error bars indicated standard deviations of 3 separate measurements.

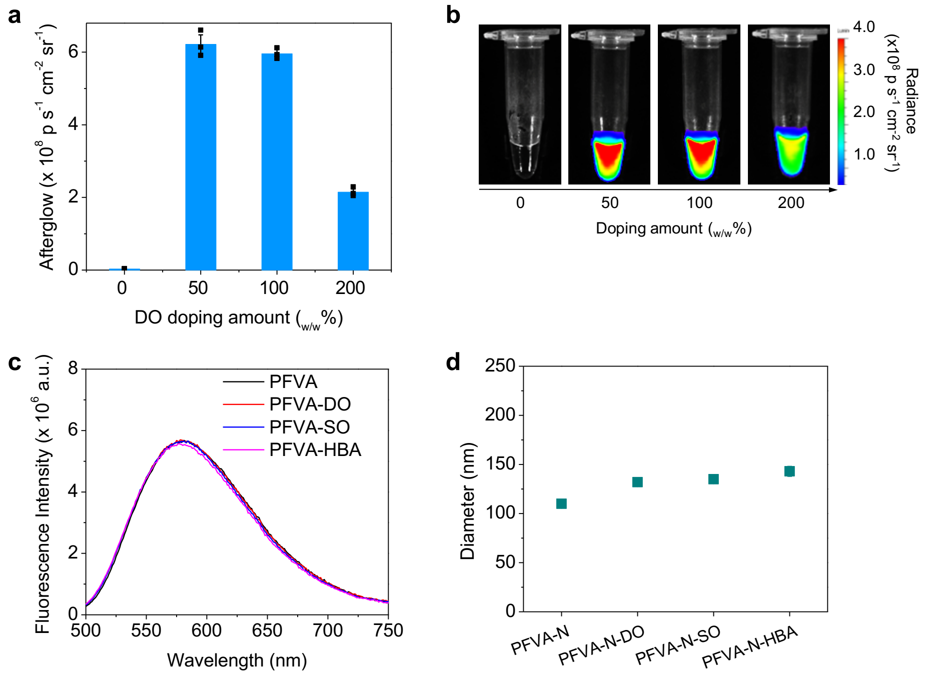

Supplementary Figure 10. Optimization of afterglow substrates doping ratio in 1 × PBS buffer. (a) Afterglow intensities of PFVA-N-DO ALNPs with different DO doping ratios after 808 nm laser irradiation (1 W cm^{-2}) for 5 s. [NCBS] = 0.75 μg mL^{-1} (2.5 $_{w/w}$%), [PFVA] = 30 μg mL^{-1}. (b) Afterglow images of (a) captured after laser irradiation. (c) Fluorescence spectra of PFVA ALNP nanoparticles doped with 50 $_{w/w}$% DO, SO, or HBA. Excitation: 450 nm. Emission: 500-750 nm. [PFVA] = 5 μg mL^{-1}. (d) Dynamic light scattering (DLS) profiles of PFVA-N ALNPs doped with 50 $_{w/w}$% DO, SO, or HBA. Error bars indicated standard deviations of 3 separate measurements.

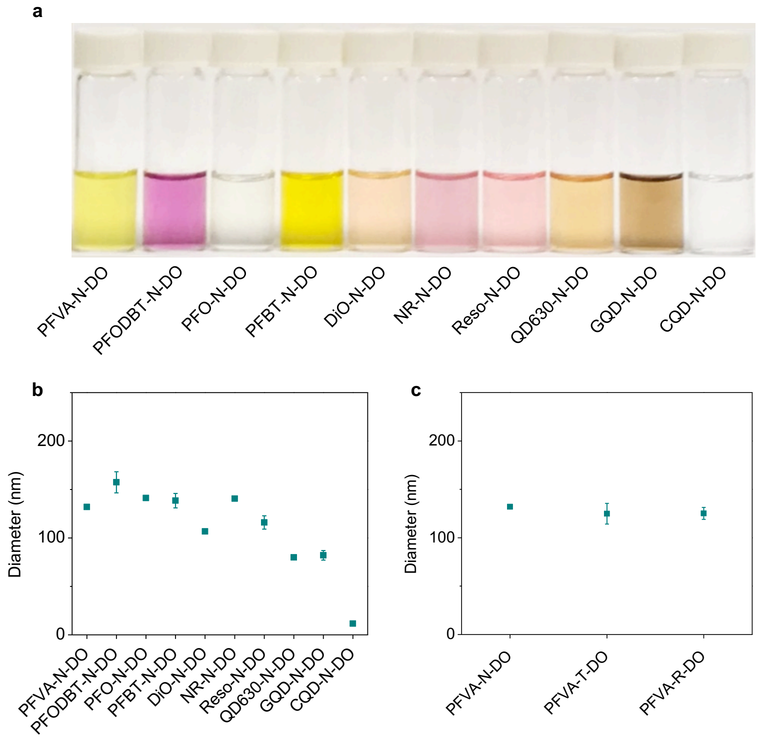

Supplementary Figure 11. (a) Photos of NCBS-DO based ALNP solutions in 1 × PBS buffer. (b-c) DLS results of (b) NCBS-DO based ALNPs and (c) PFVA-DO based ALNPs in 1 × PBS buffer.

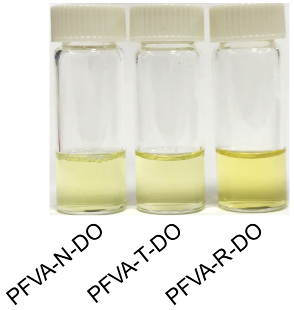

Supplementary Figure 12. Photos of PFVA-N-DO, PFVA-T-DO, and PFVA-R-DO ALNPs in 1 × PBS buffer. [PFVA] = 5 μg mL^{-1}, [DO] = 2.5 μg mL^{-1}, [NCBS], [TPP], or [RB] = 0.125 μg mL^{-1}.

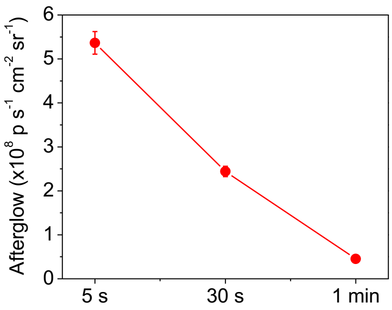

Supplementary Figure 13. Optimization of laser irradiation time for ALNPs in 1 × PBS buffer. Afterglow luminescence intensities of PFVA-N-DO ALNPs ([NCBS] = 0.75 μg mL^{-1}, [DO] = 15 μg mL^{-1}, [PFVA] = 30 μg mL^{-1}) were obtained after irradiation of 808 nm laser at 1 W cm^{-2} for different time periods. Error bars indicated standard deviations of 3 separate measurements.

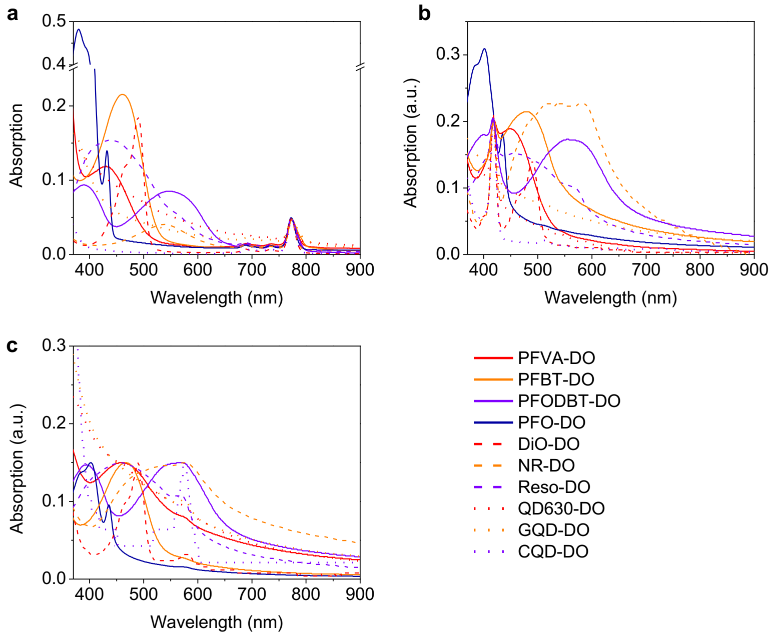

Supplementary Figure 14. Normalized absorption spectra of (a) NCBS, (b) TPP, and (c) RB - DO based ALNPs in 1 × PBS buffer.

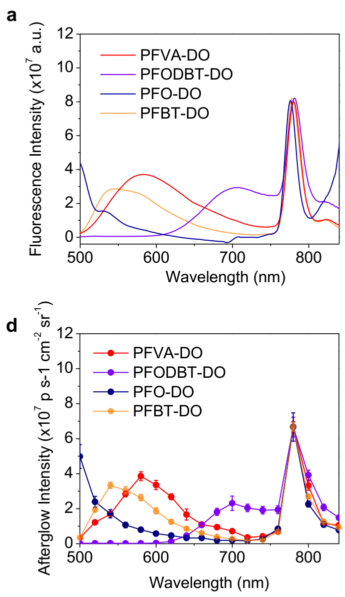

Supplementary Figure 15. Normalized (a-c) fluorescence and (d-f) afterglow spectra of NCBS-DO based ALNPs of varying afterglow relay units in 1 × PBS buffer. Fluorescent excitation: 450 nm. Afterglow spectra were captured after light illumination (1 W cm^{-2} 808 nm laser) for 5 s. Error bars indicated standard deviation of triplicate measurements.

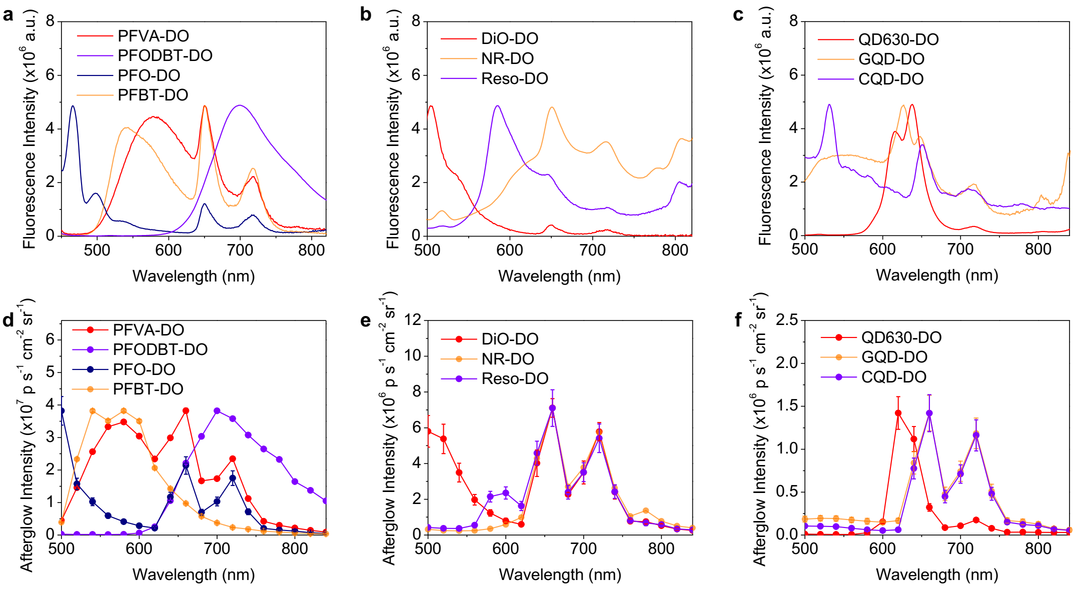

Supplementary Figure 16. Normalized (a-c) fluorescence and (d-f) afterglow spectra of TPP-DO based ALNPs of varying afterglow relay units in 1 × PBS buffer. Fluorescent excitation:

450 nm. Afterglow spectra were captured after light illumination (0.1 W cm^{-2} white light) for 5 s. Error bars indicated standard deviation of triplicate measurements.

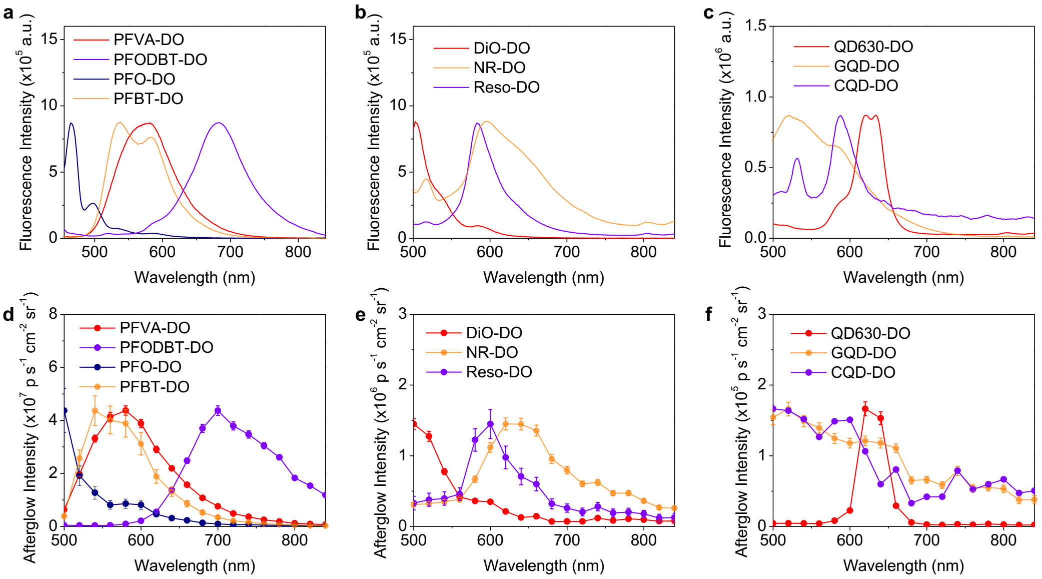

Supplementary Figure 17. Normalized (a-c) fluorescence and (d-f) afterglow spectra of RB-DO based ALNPs of varying afterglow relay units in 1 × PBS buffer. Fluorescent excitation: 450 nm. Afterglow spectra were captured after light illumination (0.1 W cm^{-2} white light) for 5 s. Error bars indicated standard deviation of triplicate measurements.

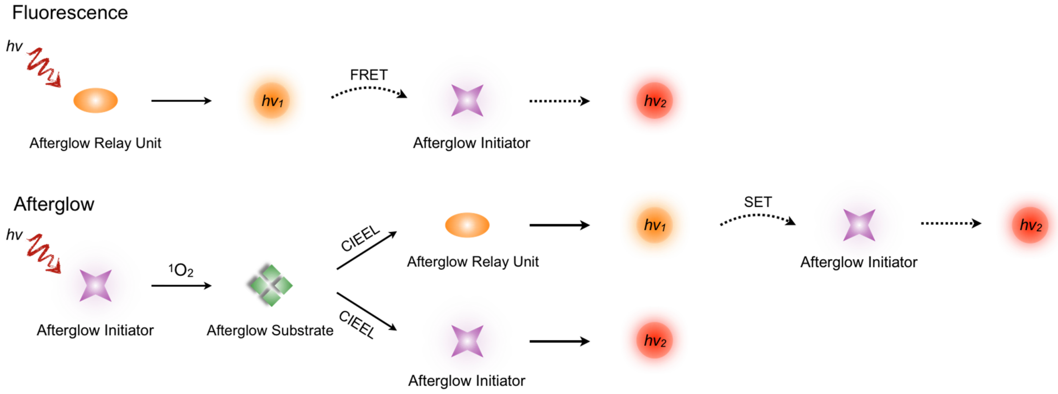

Supplementary Figure 18. Comparison of fluorescence and afterglow process in ALNPs.

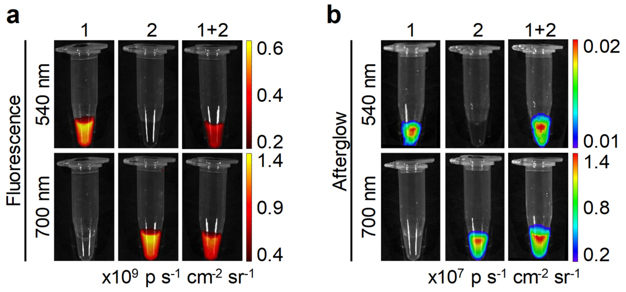

Supplementary Figure 19. Multiplexed imaging using PFBT-N-DO and PFODBT-N-DO. (a) Fluorescence images (1) PFBT-N-DO, (2) PFODBT-N-DO, and (3) 1:1 mixture of PFBT-N-DO and PFODBT-N-DO. Fluorescence images were captured with excitation/emission at 465/540 nm and 605/700 nm, respectively. (b) Afterglow imaging of (1) PFBT-N-DO, (2) PFODBT-N-DO, and (3) 1:1 mixture of PFBT-N-DO and PFODBT-N-DO at 540 and 700 nm, respectively. Afterglow images were captured after 808 nm laser irradiation (1 W cm^{-2}) for 5 s. [PFBT] = [PFODBT] = 15 μg mL^{-1}, [NCBS] = 0.375 μg mL^{-1}, [DO] = 7.5 μg mL^{-1}.

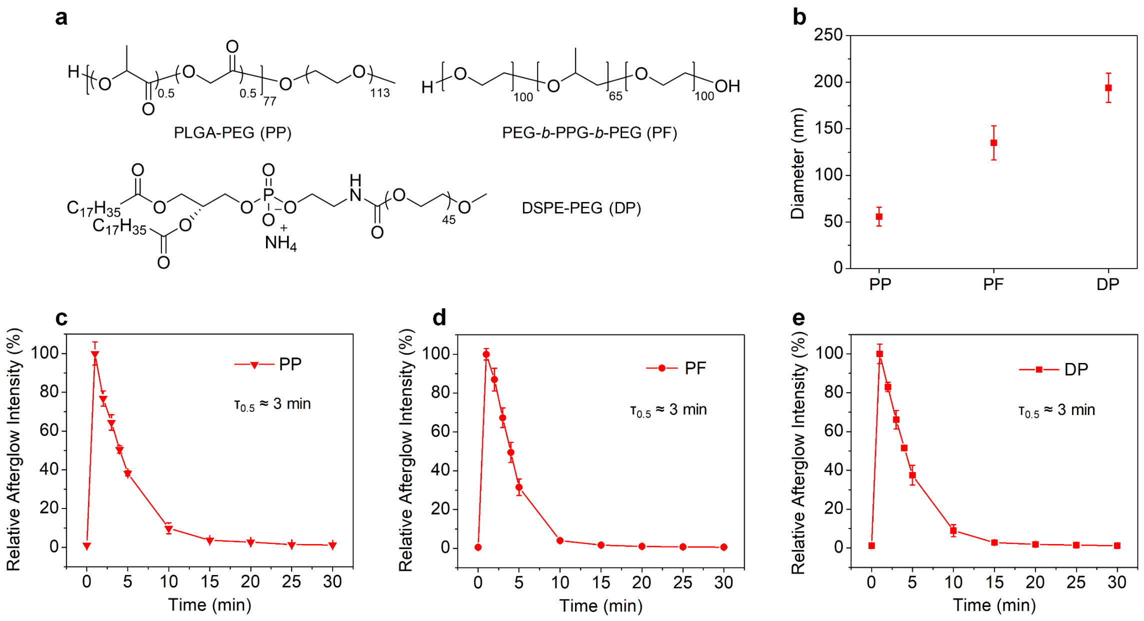

Supplementary Figure 20. Decay of afterglow luminescence of ALNPs. (a) Chemical structures of the amphiphilic polymers to prepare ALNPs of different sizes. (b) DLS profiles of PFVA-N-DO ALNPs prepared via different amphiphilic polymers (PP, PF, and DP). (c-d) Afterglow luminescence decay curves of PFVA-N-DO ALNPs prepared via PP (c), PF (d), and

DP (e). [PFVA] = 3 μg mL^{-1}, [NCBS] = 0.075 μg mL^{-1}, [DO] = 1.5 μg mL^{-1}. Afterglow luminescence was recorded after 808 nm laser (0.1 W cm^{-2}) irradiation for 5 s. Error bars indicated standard deviation of triplicate measurements.

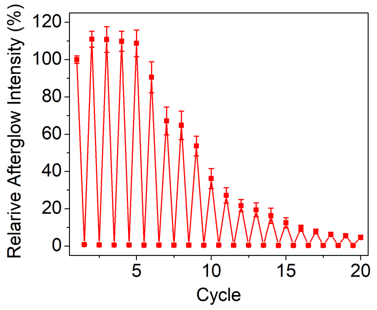

Supplementary Figure 21. Relative afterglow intensity of PFVA-N-DO ALNPs ([PFVA] = 30 μg mL^{-1}, [NCBS] = 0.75 μg mL^{-1}, [DO] = 15 μg mL^{-1}) after laser irradiation (808 nm, 0.3 W cm^{-2}) followed by cessation for 20 cycles. Error bars indicated standard deviation of triplicate measurements.

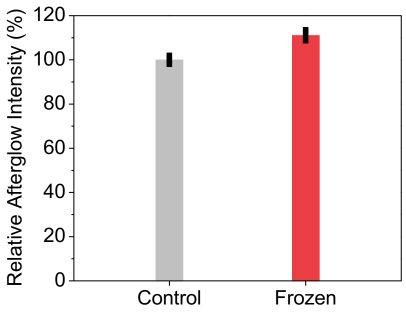

Supplementary Figure 22. Relative afterglow luminescence of PFVA-N-DO ALNPs measured immediately after 808 nm laser irradiation (Control) or thawy PFVA-N-DO ALNPs pre-irradiated with laser followed by storage at -20 °C for 48 h (Frozen). [PFVA] = 30 μg mL^{-1}, [NCBS] = 0.75 μg mL^{-1}, [DO] = 15 μg mL^{-1}; laser irradiation: 808 nm, 0.3 W cm^{-2}. Error bars indicated standard deviation of triplicate measurements.

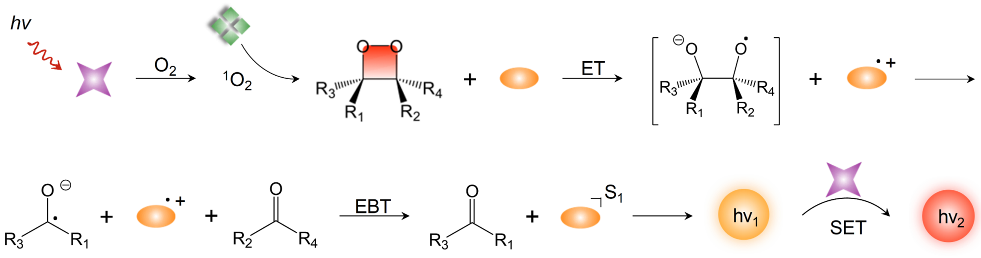

Supplementary Figure 23. Schematic illustration of CIEEL mechanism in generic afterglow approach. ET, electron transfer; EBT, electron back transfer.

Compound	Chemical Structure	HOMO	LUMO	E_{HOMO} (eV)	E_{LUMO} (eV)
DO-IMD	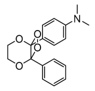	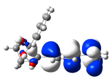	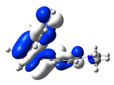	-5.16	-0.41
SO-IMD	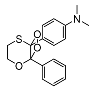	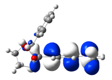	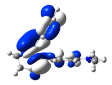	-5.14	-0.58
HBA-IMD	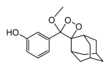	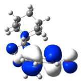	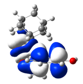	-6.19	-0.67
Reso	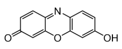	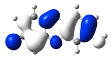	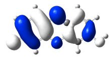	-5.94	-2.86

Supplementary Figure 24. Summary of calculated HOMOs and LUMOs of DO/SO/HBA-IMD and Reso by Gaussian 09 software based on density functional theory (DFT) with B3LYP/6-31G(d) method.

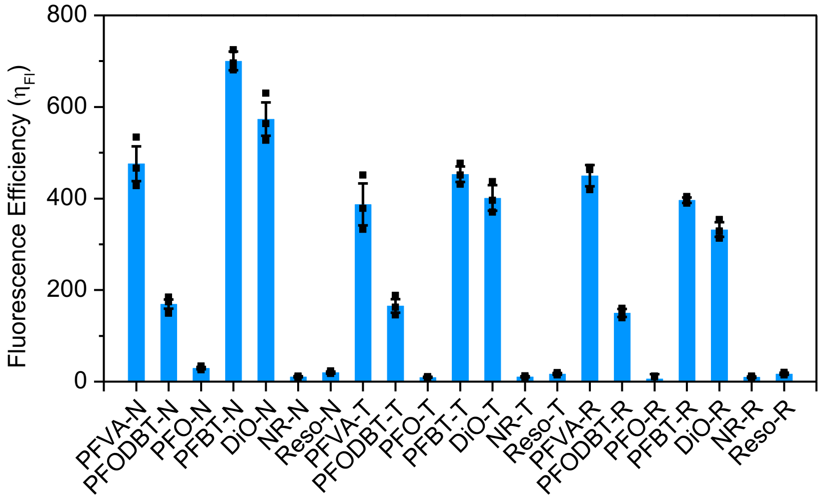

Supplementary Figure 25. Fluorescence efficiencies (η_{Fl}) of ALNPs ([afterglow relay unit] = 5 μg mL^{-1}, [afterglow initiator] = 0.125 μg mL^{-1}) without afterglow substrates in 1 × PBS buffer. Error bars indicated standard deviation of triplicate measurements.

a

Model Summary[b]

Model	R	R Square	Adjusted R Square	Std. Error of the Estimate	Durbin-Watson
1	.971[a]	.944	.936	.64955	1.760

a. Predictors: (Constant), EHOMO, LnΦCl, LnηFl, LnΦS1

b. Dependent Variable: LnΦAfterglow

b

ANOVA[a]

Model		Sum of Squares	df	Mean Square	F	Sig.
1	Regression	211.555	4	52.889	125.352	.000[b]
	Residual	12.658	30	.422		
	Total	224.213	34			

a. Dependent Variable: LnΦAfterglow

b. Predictors: (Constant), EHOMO, LnΦCl, LnηFl, LnΦS1

c

Coefficients[a]

Model		Unstandardized Coefficients B	Std. Error	Standardized Coefficients Beta	t	Sig.	Collinearity Statistics Tolerance	VIF
1	(Constant)	13.126	2.089		6.283	.000		
	LnΦCl	.463	.024	1.025	19.075	.000	.652	1.535
	LnΦS1	2.759	.356	.424	7.755	.000	.630	1.586
	LnηFl	.405	.067	.267	6.058	.000	.972	1.029
	EHOMO	2.696	.348	.339	7.751	.000	.984	1.016

a. Dependent Variable: LnΦAfterglow

d

Collinearity Diagnostics[a]

Model	Dimension	Eigenvalue	Condition Index	Variance Proportions (Constant)	LnΦCl	LnΦS1	LnηFl	EHOMO
1	1	4.631	1.000	.00	.01	.00	.00	.00
	2	.259	4.225	.00	.63	.00	.05	.00
	3	.085	7.389	.00	.04	.02	.94	.00
	4	.023	14.106	.01	.31	.92	.00	.02
	5	.002	55.504	.99	.01	.06	.00	.97

a. Dependent Variable: LnΦAfterglow

e

Residuals Statistics[a]

	Minimum	Maximum	Mean	Std. Deviation	N
Predicted Value	-4.0644	6.0585	1.0151	2.49444	35
Residual	-1.41245	1.62062	.00000	.61015	35
Std. Predicted Value	-2.036	2.022	.000	1.000	35
Std. Residual	-2.174	2.495	.000	.939	35

a. Dependent Variable: LnΦAfterglow

f

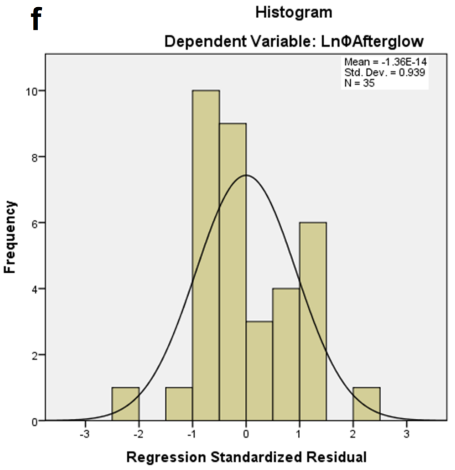

g

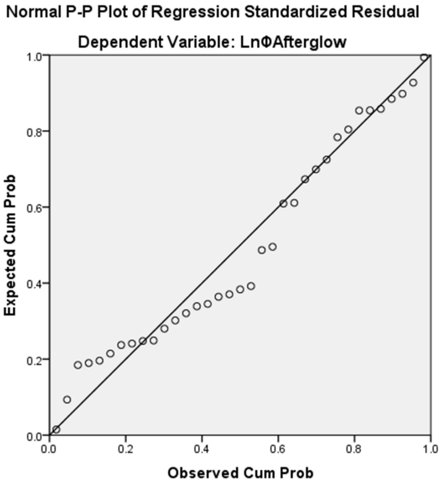

h

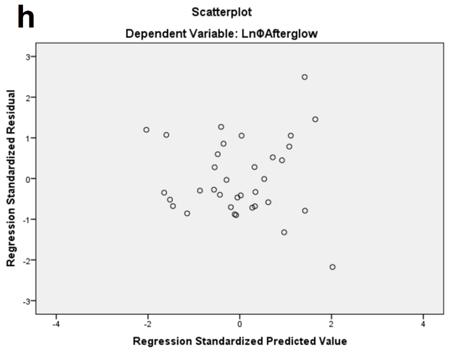

Supplementary Figure 26. Statistic results of supervised machine learning using linear regression algorithm to analyse modified descriptors by SPSS software. Original descriptors and $\Phi_{Afterglow}$ were transformed to modified descriptors Ln(Φ_{Cl}), Ln(Φ_{S1}), Ln(η_{Fl}), and Ln($\Phi_{Afterglow}$), respectively. (a) Model summary of linear regression. (b) Analysis of variance (ANOVA) of regression model. (c) Coefficients of modified descriptors. (d) Collinearity diagnostics of regression model. (e) Residual statistics of regression model. (f) Histogram of

regression standardized residual. (g) Normal P-P Plot of regression standardized residual. (h) Scatterplot of dependent variable Ln($\Phi_{Afterglow}$). The ultimate fitted equation was demonstrated as Eq.1.

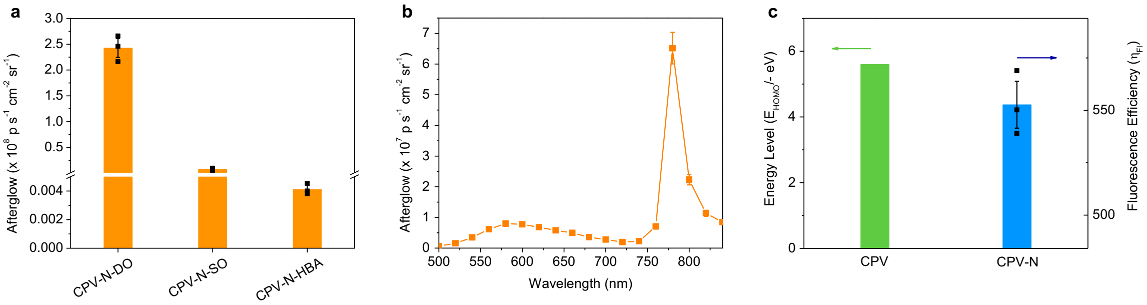

Supplementary Figure 27. (a) Afterglow intensity of CPV ALNPs ([NCBS] = 0.75 μg mL^{-1}, [DO/SO/HBA] = 15 μg mL^{-1} (50 $_{w/w}$%), [CPV] = 30 μg mL^{-1}) after irradiation by 808 nm laser at 1 W cm^{-2} for 5 s in 1 × PBS buffer. (b) Normalized afterglow spectrum of CPV-N-DO. (c) HOMO energy level of CPV and fluorescence efficiency of CPV-N ([CPV] = 5 μg mL^{-1}, [NCBS] = 0.125 μg mL^{-1}). Error bars indicated standard deviation of triplicate measurements.

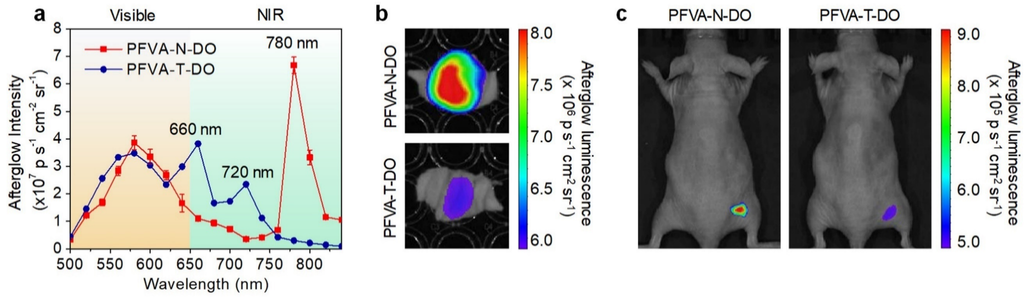

Supplementary Figure 28. Tissue penetration study of PFVA-N-DO versus PFVA-T-DO. (a) Normalized afterglow spectra of PFVA-N-DO and PFVA-T-DO. Error bars indicated standard deviation of triplicate measurements. (b) Afterglow images of PFVA-N-DO and PFVA-T-DO penetrating through mouse skin. (c) Afterglow images of PFVA-N-DO and PFVA-T-DO penetrating through a living mouse. [PFVA] = 30 μg mL^{-1}, [DO] = 15 μg mL^{-1}, [NCBS] or

[TPP] = 0.75 μg mL^{-1}. PFVA-N-DO and PFVA-T-DO was respectively irradiated with 808 nm laser and white light for 5 s. Exposure (integration) time: 1s. Error bars indicated standard deviation of triplicate measurements.

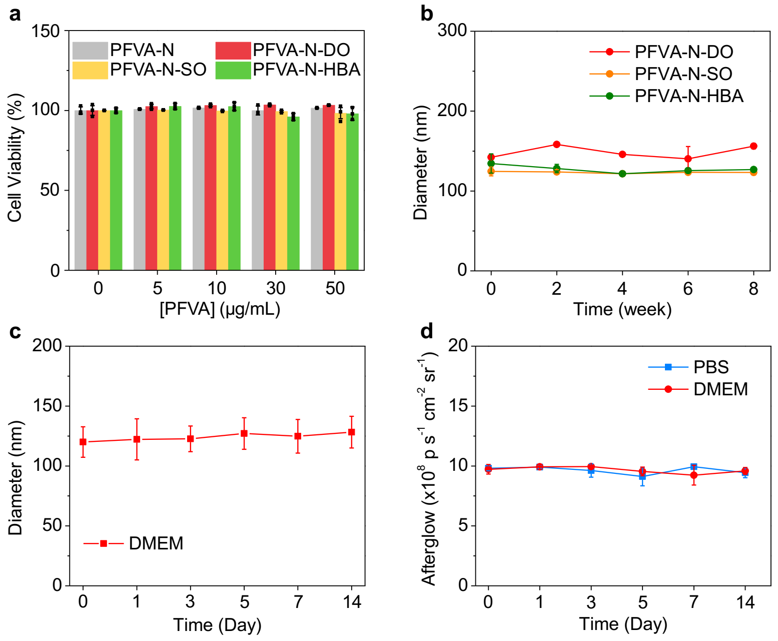

Supplementary Figure 29. Cytotoxicity and stability studies of ALNPs. (a) Cytotoxicity study of PFVA-N ALNPs. (b) Stability study of PFVA-N ALNPs in 1 × PBS. (c) Stability study of PFVA-N-DO ALNPs in cell culture medium DMEM supplemented with 10% FBS. (d) Afterglow luminescence of PFVA-N-DO ALNPs during storage in 1 × PBS or DMEM supplemented with 10% FBS. [PFVA] = 30 μg mL^{-1}, [NCBS] = 0.75 μg mL^{-1}, [DO] = 15 μg mL^{-1}. Afterglow luminescence was measured after 808 nm laser irradiation (1 W cm^{-2}) of solutions for 5 s. Error bars indicated standard deviation of triplicate measurements.

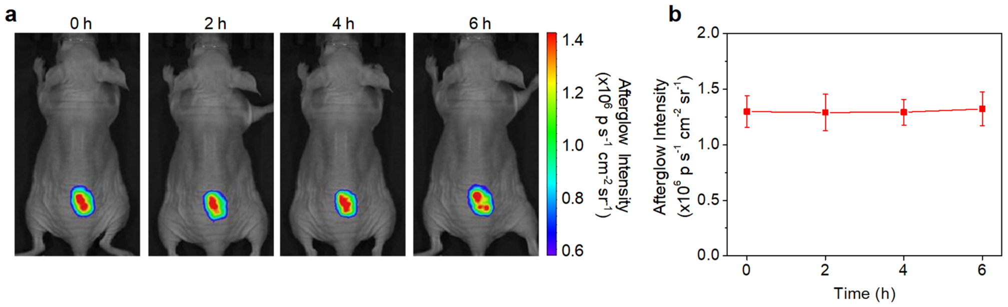

Supplementary Figure 30. Stability of PFVA-N-DO ALNPs in biological tissue. (a) Afterglow images of PFVA-N-DO at designated time points after subcutaneous injection (50 μL) into a living mouse. Afterglow images were acquired after 808 nm laser irradiation (0.3 W cm^{-2}) for 5 s. (b) Quantification of afterglow signals from the injection area. Error bars indicated standard deviation of triplicate measurements.

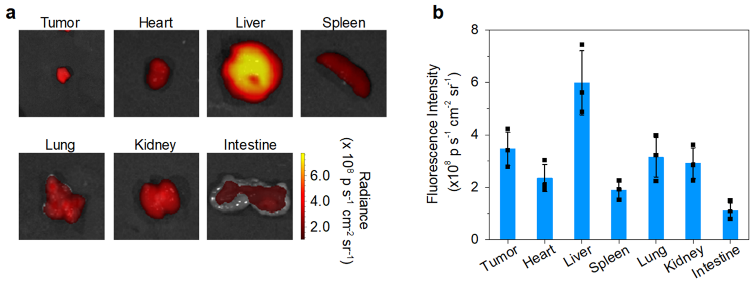

Supplementary Figure 31. Biodistribution study of in vivo tumor imaging using PFVA-N-DO ALNPs. (a) Ex vivo NIR fluorescence images and (b) quantification of NIR fluorescence intensities of tumor and major organs from 4T1 tumor-bearing mice at 6 h after intravenous injection of PFVA-N-DO (([PFVA] = 250 μg mL^{-1}, [DO] = 125 μg mL^{-1}, [NCBS] = 6.25 μg mL^{-1}, 250 μL). Excitation wavelength: 710 nm; emission wavelength: 780 nm. Error bars indicated standard deviations of 3 separate measurements (n = 3).

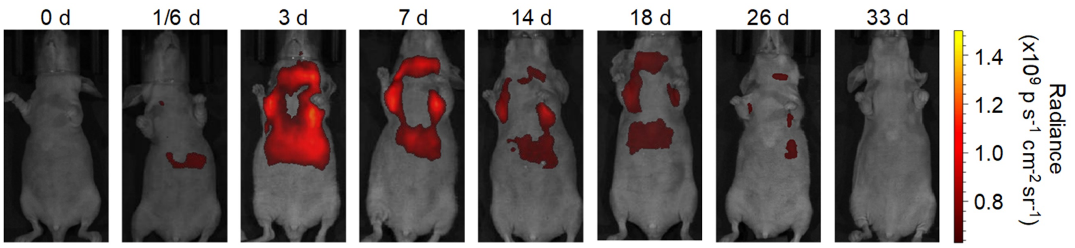

Supplementary Figure 32. Representative NIR fluorescence images of a living mouse at different time points after intravenous injection of PFVA-N-DO ([PFVA] = 250 μg mL^{-1}, [DO] = 125 μg mL^{-1}, [NCBS] = 6.25 μg mL^{-1}, 250 μL). Excitation wavelength: 710 nm; emission wavelength: 780 nm.

Supplementary Table 1. Comparison of SBRs of representative optical agents for in vivo imaging.

Modality	Materials	SBR	Application	Wavelength (nm)	Ref.
**First NIR Fluorescence** (< 1000 nm)	Antibody labelled with FITC	9.74	Imaging of intestinal immune cells in patients	205-585 nm	2
	iRFP (fluorescent protein)	4.0-4.5 (7.0 mm deep)	Deep tissue imaging	740 nm	3
	iRFP720	~ 10 (7.0 mm deep)	Deep tissue imaging	760 nm	4
	RGD-labeled upconversion nanophosphor	24	Tumor imaging	800 ± 12 nm	5
	GB138 (bearing IRDye 800)	15-20	Tumor imaging	~804 nm (ICG filter)	6
**Second NIR Fluorescence** (> 1000 nm)	SBP-M13-SWNT	134	Tumor imaging	950-1400 nm	7
	IR-12N3@Erbitux	~ 10	Tumor imaing	1000-1500 nm	8
	Ag_2S quantum dot	14-16	Tumor imaging	~1200 nm	9
	CP-IRT	8.3	Tumor imaging	> 1200 nm	10
	p-FE-PTX-FA nanoparticle	20	Tumor imaging	> 1300 nm	11
	p-FE	~12	Tumor imaging	> 1300 nm	12
	CH1055-PEG	5.50	Brain tumor imaging	> 1300 nm	13
**Chemiluminescence**	L012 and hemin-Pdots	18	Imaging of endogenous ROS	470 and 540 nm	14
	Probe 2a	20	imaging of H_2O_2	690 nm	15

**Bioluminescence**	QD655-Luc8-R9	~ 1000	Mouse imaging	655 nm	16
	RET_2IR@cRGD	116	Tumor imaging	778 nm	17
**Cerenkov luminescence**	[^{18}F]FDG-excited fluorescein	154	Phantom imaging	GFP emission filter	18
	^{90}Y-1	29	Tumor imaging	-	19
	^{90}Y-$PRGD_2$	3.4	Tumor imaging	-	20
**Persistent luminescence**	LPLNP-TAT	172	Imaging of subcutaneous nanoparticle solution	694 nm	21
	PLNP	186	Imaging of subcutaneous nanoparticle solution	695 nm	22
	PEG-LPLNP	1826	Imaging of subcutaneous nanoparticle solution	695 nm	23
	ZGC-1	275	Imaging of subcutaneous nanoparticle solution	696 nm	24
**Afterglow** (This study)	PFVA-N-DO ALNP	1136 (1.5 cm deep)	Deep-tissue imaging	780 nm	-
		2922	Tumor imaging	780 nm	-

Supplementary References

1. Green O, Eilon T, Hananya N, Gutkin S, Bauer CR, Shabat D. Opening a gateway for chemiluminescence cell imaging: Distinctive methodology for design of bright chemiluminescent dioxetane probes. *ACS Cent. Sci.* **3**, 349-358 (2017).

2. Atreya R, *et al.* In vivo imaging using fluorescent antibodies to tumor necrosis factor predicts therapeutic response in Crohn's disease. *Nat. Med.* **20**, 313-318 (2014).

3. Filonov GS, Piatkevich KD, Ting L-M, Zhang J, Kim K, Verkhusha VV. Bright and stable near-infrared fluorescent protein for in vivo imaging. *Nat. Biotechnol.* **29**, 757-761 (2011).

4. Shcherbakova DM, Verkhusha VV. Near-infrared fluorescent proteins for multicolor in vivo imaging. *Nat. Methods* **10**, 751-754 (2013).

5. Xiong L, Chen Z, Tian Q, Cao T, Xu C, Li F. High contrast upconversion luminescence targeted imaging in vivo using peptide-labeled nanophosphors. *Anal. Chem.* **81**, 8687-8694 (2009).

6. Blum G, von Degenfeld G, Merchant MJ, Blau HM, Bogyo M. Noninvasive optical imaging of cysteine protease activity using fluorescently quenched activity-based probes. *Nat. Chem. Biol* **3**, 668-677 (2007).

7. Ghosh D, Bagley AF, Na YJ, Birrer MJ, Bhatia SN, Belcher AM. Deep, noninvasive imaging and surgical guidance of submillimeter tumors using targeted M13-stabilized single-walled carbon nanotubes. *Proc. Natl. Acad. Sci. USA* **111**, 13948-13953 (2014).

8. Zhu S, *et al.* Repurposing cyanine NIR-I dyes accelerates clinical translation of near-infrared-II (NIR-II) bioimaging. *Adv. Mater.* **30**, 1802546 (2018).

9. Hong G*, et al.* In vivo fluorescence imaging with Ag_2S quantum dots in the second near-infrared region. *Angew. Chem. Int. Edit.* **51**, 9818-9821 (2012).

10. Wang W*, et al.* Molecular cancer imaging in the second near-infrared window using a renal-excreted NIR-II fluorophore-peptide probe. *Adv. Mater.* **30**, 1800106 (2018).

11. Ma Z*, et al.* A theranostic agent for cancer therapy and imaging in the second near-infrared window. *Nano Res.* **12**, 273-279 (2018).

12. Wan H*, et al.* A bright organic NIR-II nanofluorophore for three-dimensional imaging into biological tissues. *Nat. Commun.* **9**, 1171 (2018).

13. Antaris AL*, et al.* A small-molecule dye for NIR-II imaging. *Nat. Mater.* **15**, 235-242 (2015).

14. Cai L, Deng L, Huang X, Ren J. Catalytic chemiluminescence polymer dots for ultrasensitive in vivo imaging of intrinsic reactive oxygen species in mice. *Anal. Chem.* **90**, 6929-6935 (2018).

15. Green O, Gnaim S, Blau R, Eldar-Boock A, Satchi-Fainaro R, Shabat D. Near-infrared dioxetane luminophores with direct chemiluminescence emission mode. *J. Am. Chem. Soc.* **139**, 13243-13248 (2017).

16. So M-K, Xu C, Loening AM, Gambhir SS, Rao J. Self-illuminating quantum dot conjugates for in vivo imaging. *Nat. Biotechnol.* **24**, 339-343 (2006).

17. Xiong L, Shuhendler AJ, Rao J. Self-luminescing BRET-FRET near-infrared dots for in vivo lymph-node mapping and tumour imaging. *Nat. Commun.* **3**, 1193 (2012).

18. Thorek DLJ, Ogirala A, Beattie BJ, Grimm J. Quantitative imaging of disease signatures through radioactive decay signal conversion. *Nat. Med.* **19**, 1345-1350 (2013).

19. Satpati D, Hausner SH, Bauer N, Sutcliffe JL. Cerenkov luminescence imaging of αvβ6 integrin expressing tumors using 90Y-labeled peptides. *J. Label. Compd. Radiopharm* **57**, 558-565 (2014).

20. Carpenter CM*, et al.* Cerenkov luminescence endoscopy: improved molecular sensitivity with β(−)-emitting radiotracers. *J. Nucl. Med.* **55**, 1905-1909 (2014).

21. Wu S-Q, Chi C-W, Yang C-X, Yan X-P. Penetrating peptide-bioconjugated persistent nanophosphors for long-term tracking of adipose-derived stem cells with superior signal-to-noise Ratio. *Anal. Chem.* **88**, 4114-4121 (2016).

22. Maldiney T*, et al.* The in vivo activation of persistent nanophosphors for optical imaging of vascularization, tumours and grafted cells. *Nat. Mater.* **13**, 418-426 (2014).

23. Abdukayum A, Chen J-T, Zhao Q, Yan X-P. Functional near infrared-emitting Cr^{3+}/Pr^{3+} co-doped zinc gallogermanate persistent luminescent nanoparticles with super long afterglow for in vivo targeted bioimaging. *J. Am. Chem. Soc.* **135**, 14125-14133 (2013).

24. Li Z*, et al.* Direct aqueous-phase synthesis of sub-10 nm "luminous pearls" with enhanced in vivo renewable near-infrared persistent luminescence. *J. Am. Chem. Soc.* **137**, 5304-5307 (2015).
